# A decade of cold Eurasian winters reconstructed for the early 19th century

**DOI:** 10.1038/s41467-022-29677-8

**Published:** 2022-04-19

**Authors:** Lukas Reichen, Angela-Maria Burgdorf, Stefan Brönnimann, Jörg Franke, Ralf Hand, Veronika Valler, Eric Samakinwa, Yuri Brugnara, This Rutishauser

**Affiliations:** Oeschger Centre for Climate Change Research and Institute of Geography, University of Bern, Bern, Switzerland

**Keywords:** Palaeoclimate, Climate change

## Abstract

Annual-to-decadal variability in northern midlatitude temperature is dominated by the cold season. However, climate field reconstructions are often based on tree rings that represent the growing season. Here we present cold-season (October-to-May average) temperature field reconstructions for the northern midlatitudes, 1701-1905, based on extensive phenological data (freezing and thawing dates of rivers, plant observations). Northern midlatitude land temperatures exceeded the variability range of the 18th and 19th centuries by the 1940s, to which recent warming has added another 1.5 °C. A sequences of cold winters 1808/9-1815/6 can be explained by two volcanic eruptions and unusual atmospheric flow. Weak southwesterlies over Western Europe in early winter caused low Eurasian temperatures, which persisted into spring even though the flow pattern did not. Twentieth century data and model simulations confirm this persistence and point to increased snow cover as a cause, consistent with sparse information on Eurasian snow in the early 19th century.

## Abstract (web summary)

A cold season climate reconstruction for the northern midlatitudes based on freezing and thawing dates of rivers shows a cold spell 1808/9-1815/6 affecting Eurasia. In addition to two volcanic eruptions, increased snow cover played an important role.

## Introduction

**Fig. 1:**
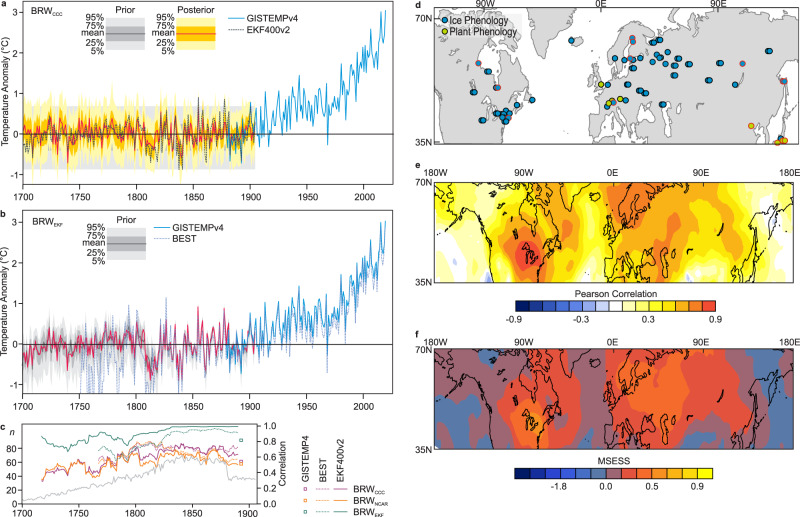
Cold-season temperatures from 1701 to 2020 over northern midlatitude land areas. **a**, **b** Time series of cold-season temperatures over land areas between 35 and 70°N from BRW_CCC_ and BRW_EKF_ (including prior and posterior; not shown for BRW_EKF_ for better visualisation) as well as BEST, and GISTEMP4. Anomalies are relative to the period of overlap (1880–1905). **c** 31-yr moving correlations between our reconstructions and other time series (only 25 years of overlap are available for GISTEMP4). The number of assimilated observations *n* is shown in grey. **d** Locations of the 68 phenological series that entered the reconstruction (black circles) and the 14 used for evaluation (red circles). Pearson correlation (**e**) and mean-squared error skill score (MSESS, **f**) of BRW_CCC_ evaluated against EKF400v2, 1851–1900.

The cold-season temperature of the northern midlatitude land areas^1–3^ shows distinct decadal variations, such as sequences of cold winters around 1880 or 1970 (Fig. 1). Studying such episodes in the past is hampered by the fact that most temperature proxies for the northern midlatitudes, such as those derived from tree rings (with some exceptions^4^), cover the growing season^5^. Some proxies, including phenological data or documentary records, do cover other parts of the year^6^ and are successfully used in regional studies^7,8^. However, they are rarely fully exploited for continental-to-global climate field reconstructions. For instance, the PAGES2k data set contains only three annually resolved documentary proxies^9^. Here we show that such series are more abundant than previously thought and their spatial and temporal coverage is such that cold-season temperature field reconstructions based exclusively on phenological data can be generated. That enables the analysis of multiannual cold spells.

Plant and ice phenological data provide temperature information for fall (leaf colouring), early winter (freezing dates), late winter (thawing dates), and spring (blossom). In the following, we define the cold season as October-May, which comprises the above-mentioned phenological phases and is largely complementary to the climate signal from tree rings. The most important database for cold-season phenology is the Global Lake and River Ice Phenology database of the National Snow and Ice Data Center^10^. These data have been used to put the recent atmospheric warming into a 150-year perspective^11,12^. Only a few records go further back than the instrumental period, including those from Lake Suwa, Japan^13^, or the Tornio river in Lapland^14^. However, freezing and break-up of rivers has been observed systematically for many rivers in the 18th and early 19th centuries^15–17^. In 1890, Eduard Brückner used the ice-free periods of Russian rivers as a proxy for large-scale temperature back to 1720 (Fig. S1)^16^. However, many of these series have since been forgotten, and only recently the corresponding book^17^, comprising observations from hundreds of sites, was imaged and made available online. Other series published in the 1970s have not made the transition to the electronic era^15^. For a comprehensive overview of the documentary climate series and corresponding literature see ref. ^18^ and the references in the Supplementary material. Note that our collection of phenological data is not complete. For instance, sources written in non-majority languages are less often used in science, which may lead to data gaps.

We digitised and processed numerous records^17^ (Fig. S1 for an example sheet) and complemented them with other ice phenology data from Europe and North America, documentary sea-ice records from Iceland and Labrador, and plant phenological records (see Methods and Table S1)^18^. All records are from north of 35 °N. In total, 82 temperature-sensitive records were selected (Supplementary Data 1), of which fourteen were left aside for independent evaluation of the reconstruction (Fig. 1d). The latter were selected to cover all regions and seasons and to keep the data set independent from the reanalysis EKF400v2^19^, which we also use in our analyses (reconstructions that also use these series were generated, see Table S2, but only used in the Supplementary material to this paper). Except for two sea-ice records, all records are given in day-of-year for leaf colouring, freeze, thaw, and blossom. Although several records extend further back in time, we focused on the period 1701–1905 as the coverage before and after that period was limited. In particular, ice phenology data from the Siberian rivers Ob, Irtysh, Yenisey, Lena, and Amur end around 1905 (Table S1).

Reconstruction was performed using Bayesian reweighting (BRW, see Methods). Forward models in the form of multiple regression models were formulated for all proxies (sometimes after transformation, see Methods), calibrated against monthly temperature observations from nearby stations^20,21^ from the 19th or early 20th centuries (Table S1). After debiasing, they were applied to two sets of climate model simulations: the atmospheric simulation ensemble CCC400^22^ from which we used 8940 preindustrial (1602–1900) years as well as the NCAR Last Millennium ensemble (NCAR-LME) comprising 13,000 preindustrial (850–1849) years^23^. The set of observations for a given year in the past was compared to all years in a set of model simulations using a distance measure. This was converted to a probability density, which can be understood as a weight. The reconstruction for that year is the weighted average of all model years, while the cumulative sum of weights yields the posterior distribution. The reconstruction using CCC400 as prior is termed BRW_CCC_ (see Table S2 for abbreviations and an overview of the approaches), the one using NCAR-LME as prior is termed BRW_NCAR_. As a third prior we used the EKF400v2 reanalysis, which is based on instrumental data, documentary data, and proxies using an off-line assimilation approach^19^. Although based on the same model data (CCC400), this data set is independent of BRW_CCC_ as there is no overlap in the documentary series used, and the BRW_CCC_ prior is time-invariant. We applied BRW to the 30-member ensemble of EKF400v2 each year (BRW_EKF_). Finally, as averaging analogues does not strictly ensure physical consistency, we also performed reconstructions based on the closest analogues for each winter (ANA_CCC_, ANA_NCAR_).

The reconstructions were evaluated using independent phenological and instrumental data (GISTEMP4^1^, CRUTEM5^2^, BEST^3^, station data^20^). Furthermore, they were compared to the ensemble mean of EKF400v2. In addition to our reconstructions and EKF400v2, we also used sea-level pressure (SLP) reconstructions for the Atlantic-European sector by Küttel. et al.^24^ for the analysis.

## Results

### Reconstructions

A comparison with instrumental records shows that the reconstructed time series of mean temperature for the region 35–70° N over land (Fig. 1 for BRW_ccc_ and BRW_EFK_, Fig. S2 for further reconstructions, figure data are in Source Data 1), agrees well with instrumental data in the overlapping period (26 years for GISSTEMP, Table S3). The overlap with BEST is longer and confirms the good agreement, although the quality of BEST deteriorates prior to ca. 1830 and variance increases. High 31-yr moving correlations are also found with EKF400v2 (ensemble mean) further back in time (Fig. 1c), even though EKF400v2 prior to ca. 1820 has almost no information from regions outside Europe (Fig. S3). Comparing BRW_EKF_ with its parent data set EKF400v2 shows that prior to ca. 1830, phenological data add information, evidenced in a decrease in correlation (i.e., the ensemble spread is sufficiently large and phenological information sufficiently strong such that the re-weighted average deviates from the unweighted average). The choice of the prior seems to have only a small influence. BRW_NCAR_ and BRW_CCC_ are highly correlated (*r* = 0.89, over the entire period, Fig. 1c). Correlations with BRW_EKF_ are somewhat lower and amount to 0.56 and 0.65 for BRW_NCAR_ and BRW_CCC_, respectively.

We compared the field reconstructions against EKF400v2 in the period 1851 to 1900 (Fig. 1e and f for BRW_CCC_, Fig. S4 for all reconstructions). The reconstructions skill is generally good except in Alaska, the US Southwest, East Siberia, and southern Central Asia. BRW_NCAR_ performs slightly better than BRW_CCC_ in terms of correlation and root mean-squared error, while BRW_CCC_ has a larger area with a positive mean-squared error skill score (MSESS); both are clearly better than ANA_CCC_ and ANA_NCAR_ in terms of all metrics. In all following plots, lines indicate where MSESS equals 0.2 to highlight where reconstructions have skill.

We further analysed correlations between reconstructions and independent station temperature^20^ and phenological data (Fig. S5). Correlations are mostly between 0.4 and 0.8, which agrees well with Fig. S4. As a further test of the climatological consistency of the reconstructions, we performed composite analyses with respect to the effects of El Niño, volcanic eruptions, and solar variability within the 1700–1905 period (Fig. S6). We found a strong response for El Niño versus La Niña similar to that found in other studies^25^. The response for maxima versus minima in the sunspot number was found to be weak, again similar to other studies^26^. Furthermore, we find a cooling after tropical or northern extratropical volcanic eruptions (see Methods). As none of these factors was explicitly included in the reconstructions, this analysis indicates that the reconstructions capture the expected large-scale response to El Niño and external forcings.

### A long-term perspective

The reconstructions allow a 320-year perspective of climate variability and change in boreal cold-season climate. Our reconstructions complement observation-based data sets such as GISTEMP4 or others backwards in time when referenced to the overlapping period. For the 35–70° N average over land we can demonstrate how much the warming since the 1970s stands out from preindustrial variability (Fig. 1). We find that already the year 1944 during the Early Twentieth-century warming^27^ was outside the upper uncertainty limit (95-percentile of the posterior) of the warmest year of the 18th and 19th century in any combination of reconstructions (BRW_CCC_, BRW_NCAR_, BRW_EKF_) and observation-based data sets (GISTEMP4, CRUTEM5, BEST). Temperatures in the most recent decade were another 1.5 °C higher than in 1944, highlighting the extremely rapid change of northern midlatitude cold-season temperature. For comparison: The standard deviation of the annual series in the 1701–1905 period is 0.22 °C in BRW_CCC_, 0.25 °C in BRW_NCAR_, 0.29 °C in EKF400v2 and 0.32 °C in BRW_EKF_.

Prior to the 20th century, our reconstruction agrees well with that of Brückner^16^ (see Fig. S2). The reconstructions show pronounced interannual-to-decadal variability, including multi-year cold spells. The most prominent of these occurred from 1808/9 to 1815/6, which is analysed in the following. Averaged anomalies (from 1851–1900) range from −0.27 to −0.61 °C (35–70° N over land) depending on the data set analysed, even −0.88 °C in BEST (Fig. 1b), which however has only sparse data coverage.

### The 1808/9–1815/6 cold spell

**Fig. 2:**
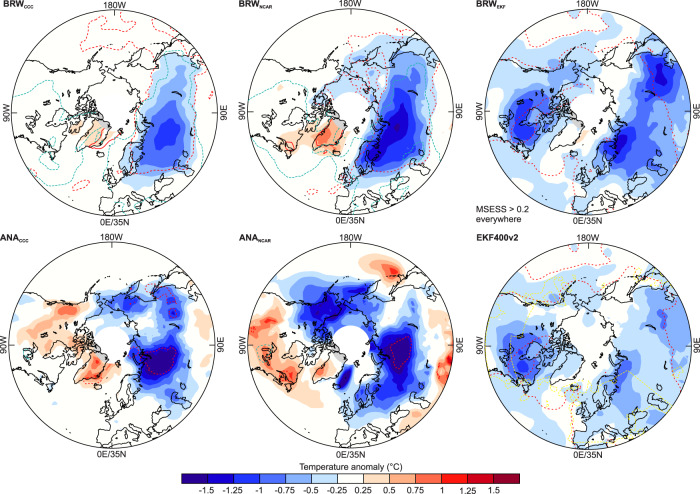
Cold-season temperature anomalies (with respect to 1851–1900) during the cold spell in the early 19th century in different reconstructions. Anomalies in 1808/9–1815/6 in BRW_CCC_, BRW_NCAR_, ANA_CCC_, ANA_NCAR_, BRW_EKF_, and EKF400v2. Dashed (−1) and solid (+1) red lines express anomalies in standard deviations of the 1851–1900 period. The green dashed lines indicate a MSESS of 0.2 relative to EKF400v2 (the yellow dashed line in EKF400 indicates an MSESS of 0.2 relative to HadCRUT5^56^, which is not spatially complete).

Composite anomaly fields of temperature for this eight-year period (Fig. 2) show that particularly cold conditions are found over Western Russia and Western Siberia. The most widespread cooling is found in BRW_EKF_, which has the information from both, station data and phenology. In regions that are not covered by phenological observations, BRW_CCC_ and BRW_NCAR_ are relaxed towards climatology. There, the closest analogues (ANA_CCC_ and ANA_NCAR_) show more variability (Fig. 2), but it should be noted that they have very little skill (MSESS lower than 0.2 almost everywhere). All data sets agree well over Eurasia. The westernmost part of the cold Eurasian region is covered by measurements and the temperature gradient across Europe also appears in instrumental-only composites^28^.

The agreement is worse over eastern Canada, where BRW_CCC_ and ANA_CCC_ are neutral, BRW_NCAR_ and ANA_NCAR_ even warm, but EKF400v2 cold. The latter is partly due to the sea-surface temperatures used in constraining EKF400v2 (Fig. S7b), and partly due to assimilated observations (Fig. S7c, note that EKF400v2 makes use of the covariance structure only up to a distance of 1500 km, so only regional observations can have an influence). A recent coupled ocean-atmosphere reconstruction^29^, though for annual means, shows cooling over North America, but less than in EKF400v2 (Fig. S7d). A recent sea-surface temperature reconstruction^30^ (Fig. S7e) shows warming off the coast of Newfoundland, perhaps supporting BRW_CCC_ and BRW_NCAR_ more than EKF400v2. Therefore we focus on Eurasia in the following. Furthermore, we address intra-seasonal variability, as early and late winter may behave differently.

In the phenology-based reconstructions, the cold period begins just around the date of the unknown 1808/9 eruption^28,31^ and ends with 1815/6, i.e., the cold season following the 1815 Tambora eruption. In fact, the period coincides with a decade-long, year-round, global cold period which is mostly attributed to a sequence of volcanic eruptions^32–34^. Previous analyses have focused mostly on boreal summer. Here we add boreal winter to the picture and study climate mechanisms during the cold season.

To address the role of atmospheric circulation we analysed SLP in EKF400v2, in the reanalysis 20CRv3^35^, in the reconstruction by Küttel et al.^24^ (Dec-May) and additionally reconstructed SLP fields for ANA_CCC_, BRW_CCC_, XBRW_CCC_ (same as BRW_CCC_, but including also those series that were left out for evaluation, see Table S2) and BRW_EKF_. This was achieved by applying the weights to SLP fields in the corresponding data sets (Fig. S8). All reconstructions show positive anomalies over northern Eurasia, but they differ over North America and the western North Atlantic. Phenological data do not seem to constrain SLP well: ANA_CCC_ has no skill at all, and in BRW_CCC_ and XBRW_CCC_ skill is low and limited to Europe and parts of North America. SLP over the Arctic in 20CRv3 in these very early years seems to be biased relative to 1851–1900. Restricting our analysis to Europe (yellow square in Fig. S8) we find a better agreement between the data sets. Here, all seven data sets show negative anomalies in the southwest and positive in the northeast. In the following, we show SLP results from EKF400v2 and Küttel et al. which allow later subseasonal analyses.

**Fig. 3:**
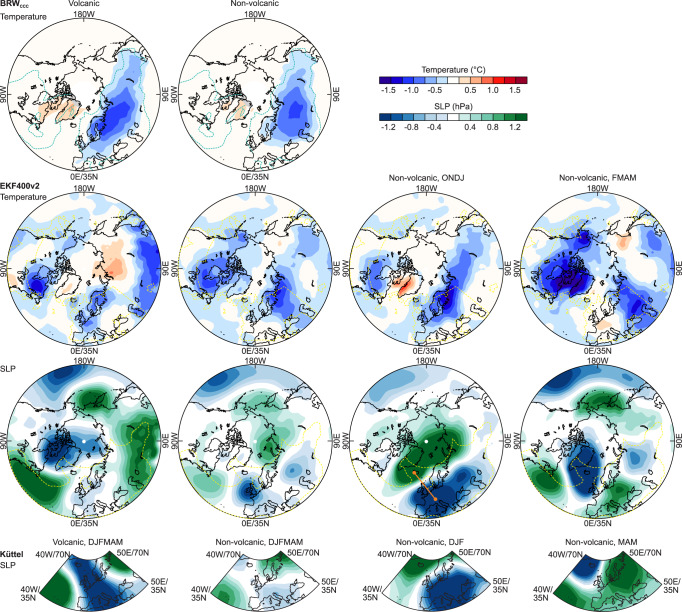
Volcanic effects and internal variability. Temperature and SLP anomalies (with respect to 1851–1900) in (left) the two cold seasons following volcanic eruptions and (second from left) the remaining 6 years of the period in BRW_CCC_ and EKF400v2. For the latter composite, anomalies are also shown for Oct-Jan and Feb-May separately. For SLP we also show composites from Küttel et al.^24^ for Dec-May, Dec-Feb, and Mar-May. The green lines indicate a MSESS of 0.2 relative to EKF400 (the yellow dashed line in EKF400 indicates an MSESS of 0.2 relative to HadCRUT5^56^ and HadSLP2^57^). The thick orange line illustrates the index defined.

For further analyses, we separated the period into cold seasons following volcanic eruptions (1809/10 and 1815/6) and others, as volcanic eruptions have an additional direct cooling effect and might trigger dynamical responses that differ from other years (Fig. 3)^36^. The volcanic years (though only two) exhibit a similar temperature pattern as the composites for all tropical eruptions (Fig. S6). In the SLP fields, they show signs of a strengthened polar vortex in EKF400v2, as expected from the literature^36^.

The superposition of radiative and dynamical effects after volcanic eruptions might be a challenge for the reconstruction. It is expected to represent both effects by overly weighting volcanic years in the prior. In fact, the two volcanic years have a 22% higher contribution (average of BRW_CCC_ and BRW_NCAR_) from volcanic years (years with nonzero global aerosol optical depth^37^ plus the two subsequent years) than an average year. However, the two effects can hardly be separated. The same volcanic composite as in Fig. 3 but in a reconstruction that has only volcanic or only non-volcanic years in the prior shows only small differences (Fig. S9). While this points to the robustness of the reconstruction, actual contributions of dynamical and radiative effects, in this case, must remain open.

The non-volcanic years exhibit a similar temperature pattern as the volcanic years. In the SLP fields, they exhibit signs of a weakened polar vortex. We further stratified the non-volcanic composite in EKF400v2 into October-January and February-May (Fig. 3; for the seasonal Küttel et al. data we use Dec-Feb and Mar-May). SLP in October-January shows a weakened Icelandic low and negative anomalies over Western and Central Europe, pointing to reduced southwesterly flow. The two data sets, EKF400v2 and Küttel et al. are in good agreement. The temperature pattern, especially the low temperatures in UK and Scandinavia, is in agreement with this situation. In February-May, the SLP pattern is almost opposite to that in October-January, again with the good agreement of EKF400v2 and Küttel et al. SLP. However, although the SLP anomalies switch signs, the temperature anomaly field shows similar anomalies as in October-January.

**Fig. 4:**
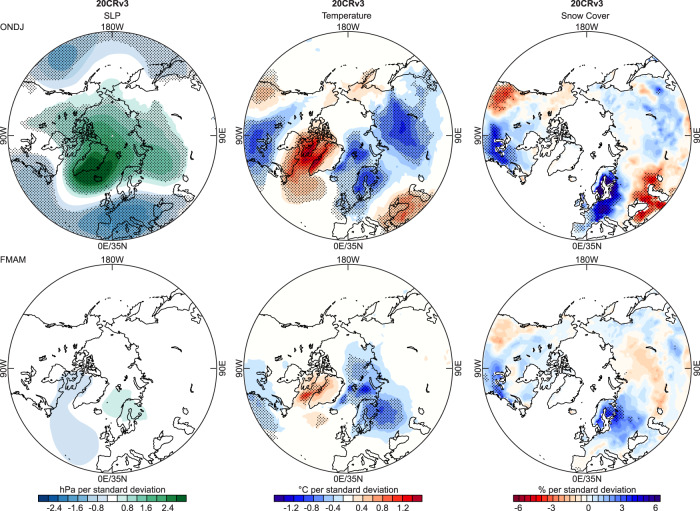
Seasonal persistence. Standardised regression coefficients of detrended SLP, temperature, and snow cover in Oct-Jan (top) and Feb-May (bottom) onto a detrended Oct-Jan SLP index (defined as the difference between two points, indicated in Fig. 3) in 20CRv3, 1901–2000. Significant (*p* < 0.05) coefficients are hatched.

We examined this phenomenon further by analysing cold seasons in the 20th century using both EKF400v2 as well as 20CRv3 (which in the 20th century is more reliable than in the early 19th century). We defined an SLP index as the difference between Iceland (22° W, 65° N) and Central Europe (8° E, 48° N, see the orange line in Fig. 3). We then detrended both the index and the analysed fields, standardised the index and regressed SLP and temperature fields for Oct-Jan and Feb-May onto the Oct-Jan SLP index (Fig. 4 for 20CRv3; Fig. S10 for EKF400v2). Results for Oct-Jan SLP show a negative Arctic Oscillation pattern, indicating a weak polar vortex. Likewise, the regression field for temperature in Oct-Jan (Fig. 4) shows the imprint of a negative Arctic Oscillation. When regressing Feb-May fields onto the Oct-Jan SLP index, we find that the SLP anomaly completely vanishes whereas the temperature anomaly persists.

### The role of snow

**Fig. 5:**
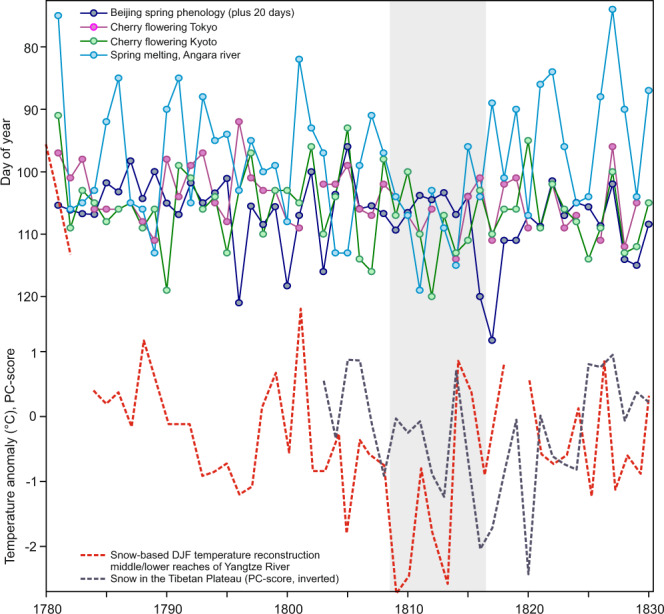
Day-of-year (note the reverse scale) of spring phenological dates from East Asia (Table S1) as well as a winter temperature reconstruction based on documentary snow data and a snow proxy from Tibet (lower scale). Note that none of these six series was used in BRW_CCC_, BRW_NCAR_, and BRW_EKF_. The grey shading indicates the period of interest.

Changes in sea ice or snow cover might help to explain the seasonal persistence of temperature anomalies. The 20CRv3 temperature regression pattern shows changes near the sea ice edge (Fig. 4), although sea ice extent in the first half of the 20th century in that data set is nearly constant. The low temperature over Western Russia is reflected in increased snow cover, which persists as a significant feature into late winter and early spring. The same analysis in a set of 36 atmospheric model simulations performed with ECHAM6 (see Methods) yields similar results (Fig. S11). The signature in albedo, including over East Asia, persists into spring, which would alter the surface energy balance. That was possibly also the case in the 1810s. Independent phenological data (not used in BRW_CCC_ and BRW_NCAR_, Table S1) from East Asia confirm that spring was unusually late and hence possibly associated with late snow disappearance (Fig. 5). In fact, two snow proxies (a temperature reconstruction for the middle and lower reaches of the Yangtze River based on snow measurements^38^ and a snow cover proxy for the Tibetan Plateau^39^) indicate high snow amounts (Fig. 5), and similar results are found for other documentary data from China^40^. The Lake Baikal region also had more snow in this period^41^, but the latter proxy (not included in Fig. 5) has a lower time resolution. Although Eurasian snow cover exhibits a high spatial variability, this implies an increase in the snow around the southern snow line in spring. Conversely, snow proxies from North America show no increase^42,43^.

Further linkages might involve the oceans. The climate system and particularly the oceans recovered only slowly from the sequence of eruptions in the early 19th century^30^. In a recent sea-surface temperature reconstruction^30^ (Fig. S6e), the period 1808/9–1815/6 is cooler than the reference period, especially in the tropics, but with warmer-than-average regions in the North Atlantic. Documentary evidence point to generally sea-ice rich summers in the Greenland Sea^44^, but with large variability. Whether the anomalous atmospheric circulation in late fall and early winter was a random realisation of internal variability, or whether the post-volcanic climate system produces such excursions more frequently, perhaps due to altered oceanic variability patterns, remains to be studied.

The multiannual cold spell during 1808/9–1815/6 was just one element of a cascade of climate effects that unfolded during the early 19th century after a sequence of volcanic eruptions, a period of a solar minimum^45^, and large internal variability. This paper presents the cold-season effects on a hemispheric scale and points to a mechanism involving snow cover, connecting cold-season effects to the next summer season. The paper also demonstrates the feasibility and the value of cold-season climate reconstructions. However, many sources of climate information remain still untapped and will help to better reconstruct past climate and thus improve our understanding of the underlying mechanisms of climatic changes.

## Methods

### Phenological data and forward modelling

For this study, we compiled a set of 82 phenological proxy series (Supplementary Data) that are sensitive to temperature in the extended cold season (October-May). Table S1 lists all series and their properties. Note that many additional series would be available. We only used series for which the forward model explained more than 20% of the variance. Further, in order not to overly distort the spatial coverage, several shorter records from New England and Northeastern Europe were not included. Of the remaining 82 series, a set of 14 was not used for the reconstruction but retained for independent revaluation, namely four series that were already assimilated in EKF400v2 (thus allowing independent comparisons) and 10 further series which we selected such as to capture all regions and sub-season (see Table S1).

The series were screened for outliers by examining values outside four standard deviations. Only a few implausible values were rejected (nine values in total, marked in Table S1). Furthermore, after applying the forward models (see next paragraph) all residuals series were tested for normality using the Shapiro-Wilk test. That led to the transformation of eight series (marked in Table S1). In all cases, optimal transformations were found by fitting distribution of the Weibull family and convert to a normal distribution.

The approach used in this paper requires the calibration of proxy forward models which can then be applied to climate model data. These proxy forward models, in the form of multiple regression models, were built using temperature data from nearby stations (see Table S1), and the climate model data were debiased with respect to the observations before applying the forward models. The station data were taken from the GHCN monthly data set^20^, supplemented with long series available from MeteoSwiss (for the case of Basel and Geneva), and previously undigitised data from the Russian empire^21^. Only monthly mean temperatures were used.

First, we considered all 8 months of the extended growing season and then restricted the months considered according to their significance (*p* < 0.1), physical consistency (sign must be correct) and time sequence (a non-significant month between two significant ones was also included). The resulting models are indicated in Table S1 (start month and end month). The models were calibrated in a preindustrial period of 30–100 years in length. The exact period was dictated mainly by the availability of station data and the often short overlap (as many of the phenological series stop in the late 19th century). Table S1 lists all calibration periods. All correlations between the phenological series and the calibrated forward models are shown in Table S1. They spread between 0.45 and 0.87 with an average of 0.67.

### Climate model data

Our approach makes use of climate model simulations as a prior. In this study, we use two sets of simulations. The first is a 30-member ensemble of atmospheric model simulations CCC400 (Chemical Climate Change over the past 400 years)^20^, which was used in several previous assimilation approaches^46^. The simulations were performed with the ECHAM5.4 model^47^ at a configuration of T63L31, corresponding to a grid spacing of 1.875°. The simulations cover the period 1600 to 2005 in an initial condition ensemble.

The model was forced with reconstructed sea-surface temperatures taken from Mann et al.^48^, but augmented with ENSO-dependent intra-annual variability^22^ and HadISST1 climatological sea ice^49^. An error in the specification of the land surface^44^ leads to summer temperature biases over some regions of Eurasia and North America in the 20th century, but the error is small in the cold season and in earlier centuries.

The second set of simulations was an ensemble of 13 all forcings simulations in the NCAR Last Millennium Ensemble, covering 850–1849^23^. Simulations were forced using the PMIP protocol^50^.

For analysing the effect of snow, atmospheric model simulations were performed with ECHAM6^51^ at a resolution of T63L47. An ensemble of 36 simulations was performed starting in 1851. In addition to initial conditions, the simulations differed in sea-surface temperatures. We used the 10 ensemble members of HadISST2^52^ sea-surface temperature data as boundary conditions, from which we constructed further members through linear combination; only one realisation was however available for sea ice^52^. All other forcings (land surface, volcanic aerosols, tropospheric aerosols, and greenhouse gas concentrations) followed the PMIP protocol^50^.

### Bayesian reweighting

In this paper, we use an analogue resampling (for another analogue approach see Neukom et al.^53^), which we term Bayesian reweighting (see also Labbé et al.^54^). Let *x* be the desired quantity (e.g., temperature) that we want to reconstruct. Bayesian approaches require specifying a prior probability of this quantity, *P*(*x*). Given a phenological observation *y* in year *j*, Bayes’ theorem reads:

1 $$P(x{{{{{\rm{|}}}}}}{y}_{j})=P({y}_{j}{{{{{\rm{|}}}}}}x)P(x)/P({y}_{j})$$

In our approach we use a large number of model years (all preindustrial years) from an ensemble simulation as prior. These are gridded data, and the reconstruction will have the same format (grid size, variables, etc.) as the prior. Each model year can be considered equally likely, and the prior is the same for each year in the past. The probability of the observation given the prior, $P({y}_{j}{|x})$, requires that phenological observations can be simulated from *x* using a forward model *H*(*x*). The denominator $P({y}_{j})$ can be expanded. For a given model year *i* (if the elements are mutually disjoint and span the full sample space), the Bayes theorem can be written as:

2 $$P({x}_{i}{{{{{\rm{|}}}}}}{y}_{j})=P({y}_{j}{{{{{\rm{|}}}}}}{x}_{i})P({x}_{i})/{\sum }_{i}P({y}_{j}{{{{{\rm{|}}}}}}{x}_{i})P({x}_{i})$$

because all *x*_*i*_ are equally likely, *P*(*x*_*i*_) = *const*. and the equation simplifies to:

3 $$P({x}_{i}{{{{{\rm{|}}}}}}{y}_{j})=P({y}_{j}{{{{{\rm{|}}}}}}{x}_{i})/{\sum }_{i}P({y}_{j}{{{{{\rm{|}}}}}}{x}_{i})$$

The expectation value of (3) is

4 $$E(x{{{{{\rm{|}}}}}}{y}_{j})={\sum }_{i}P({x}_{i}{{{{{\rm{|}}}}}}{y}_{j})\cdot {x}_{i}$$

Thus, the desired reconstruction of *x* for year *j*, *x*_*rec,j*_, is a weighted average of all *x*_*i*_:

5 $${x}_{{rec},j}=\frac{{\sum }_{i}{w}_{i,j}\cdot {x}_{i}}{{\sum }_{i}{w}_{i,j}}$$

$P({y}_{j}|{x}_{i})$ and hence the weight of a model year *x*_*i*_ depends on the difference between observed and modelled phenological data, ${y}_{j}-H\left({x}_{i}\right)$, which needs to be condensed into a distance measure *D*_*i,j*_. The weight *w*_*i,j*_ thus needs to be defined such that it maximises for *D*_*i,j*_ = 0 and decays if the differences get larger. Calculating conditional probabilities $P({y}_{j}|{x}_{i})$ directly is difficult not only because we have few instrumental observations, but mostly because overlaps between series are short or non-existent. Many combinations of documentary series are unique, i.e., no other year than the target year shares the same combination of available records. To obtain a distance measure over a set of series, we used the Euclidian distance (note that a statistical distance such as the Mahalanobis distance cannot be calculated directly due to insufficient overlap and sparse station data), thus:

6 $${D}_{{ij}}=\sqrt{\big({y}_{j}-H\big({x}_{i}\big)\big){R}^{-1}{\big({y}_{j}-H\big({x}_{i}\big)\big)}^{T}}$$

where R is the diagonal matrix of the variances of the residuals in the calibration. The weight is then obtained from converting this distance into a folded normal distribution: ${w}_{i,j}=f({D}_{i,j}|0,{\sigma }^{2})$.

In this conversion, σ depends on the number of series *n*. Applying the forward models to EKF400v2 gives some indications of this relation. Fitting χ^2^ distributions to the distances for all occurring combinations with more than 10 cases (in order to estimate the degrees of freedom) we empirically found a relation of σ ~ *n*^0.25^. The scaling factor in this relation was calibrated. The smaller the factor is chosen, the larger the variance of the reconstruction, but typically at the expense of a lower correlation. Based on testing different factors (Fig. S12) we chose a factor of 0.8, i.e., σ = 0.8 *n*^0.25^ (or σ^2^ = 0.64 *n*^0.5^).

While the approach is similar to other analogue approaches such as Neukom et al.^53^, there are some differences. For instance, we do not convert proxies to temperature for selecting the analogues but use a forward model that converts modelled monthly temperature into, e.g., a day-of-year of ice break-up and base our distance measure on that. Furthermore, Neukom et al. use the five closest analogues whereas we use weighting.

### Analysis of ENSO, solar, and volcanic forcing

For the analysis of ENSO, we used the years listed in Brönnimann et al.^55^ without those marked as volcanically perturbed. For the solar composite, we used the sunspot number (http://www.sidc.be/silso/datafiles) of the previous year and then defined the upper and lower terciles of the period 1700–1905. Finally, the volcanic composite is based on the eruptions listed in Sigl et al.^37^ (their Fig. 3). We analysed the years following the eruptions.

## Supplementary information

Supplementary Information

Description of Additional Supplementary Files

Supplementary Data 1

Supplementary Code 1

## Data Availability

The documentary data generated in this study are attached as Supplementary Data 1, the time series shown in Fig. 1 and S2 are given as Source Data 1. The field reconstructions generated in this study are available from PANGAEA, https://doi.pangaea.de/10.1594/PANGAEA.934288 ECHAM6 simulation data are available from: https://boris.unibe.ch/156682 The CCC400 model data are available from Franke et al.^46^ (http://cera-www.dkrz.de/WDCC/ui/Compact.jsp?acronym=EKF400_Input_Data, accessed 28 March 2022). The NCAR-LME simulations can be downloaded from https://www.earthsystemgrid.org/ (accessed 28 March 2022). GHCN monthly, version 4, is available from https://www.ncdc.noaa.gov/ghcn-monthly (accessed 28 March 2022). GISTEMPv4 is available from GISTEMP Team, 2021: *GISS Surface Temperature Analysis (GISTEMP), version 4*. NASA Goddard Institute for Space Studies. Data set accessed 20YY-MM-DD at https://data.giss.nasa.gov/gistemp/ (accessed 28 March 2022). CRUTEM5 is available from https://crudata.uea.ac.uk/cru/data/temperature/ (accessed 28 March 2022). BEST is available from http://berkeleyearth.org/ (accessed 28 March 2022). Sunspot numbers are available from https://wwwbis.sidc.be/silso/datafiles (accessed 28 March 2022). 20CRv3 is available from https://portal.nersc.gov/project/20C_Reanalysis/ (accessed 28 March 2022). Source data are provided with this paper.

## Source data

Source Data

## Code availability

The reconstruction code is available in the Supplementary material as Supplementary Code 1.
