# Supplementary material for: A decade of cold Eurasian winters reconstructed for the early 19th century
Source: Nat Commun. 2022 Apr 19;13:2116. doi: 10.1038/s41467-022-29677-8 (PMC9019108; doi:10.1038/s41467-022-29677-8)
Supplement: Supplementary file 1 — Supplementary Information [file 41467_2022_29677_MOESM1_ESM.pdf]

A Decade of Cold Eurasian Winters Reconstructed for the Early 19th Century

Lukas Reichen, Angela-Maria Burgdorf, Stefan Brönnimann, Jörg Franke, Ralf Hand, Veronika Valler, Eric Samakinwa, Yuri Brugnara and Matthias Rutishauser

Oeschger Centre for Climate Change Research and Institute of Geography, University of Bern, Switzerland

Supplementary Material

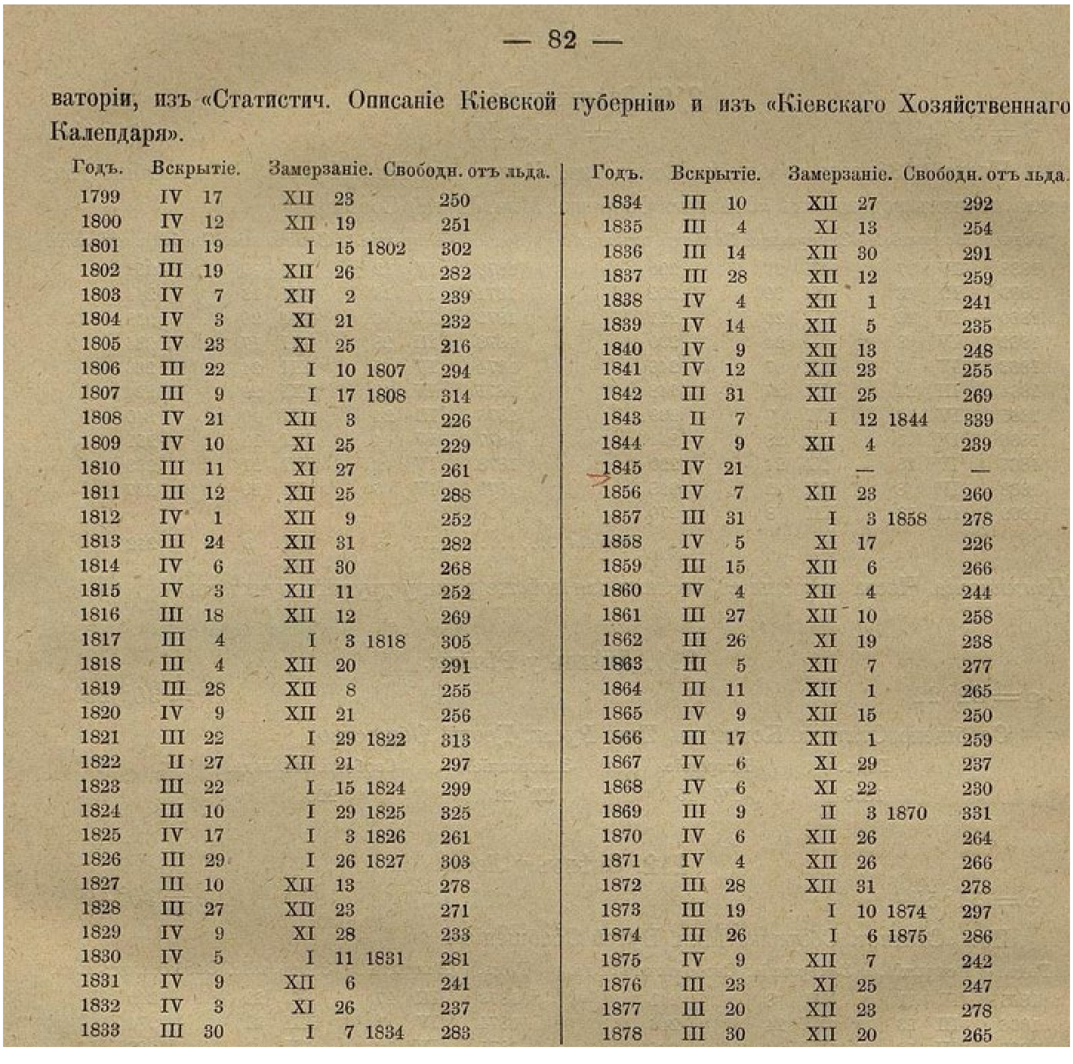

— 82 —

ваторіи, изъ «Статистич. Описаніе Кіевской губерніи» и изъ «Кіевскаго Хозяйственнаго Календаря».

Годъ.	Вскрытіе.	Замерзаніе.	Свободн. отъ льда.	Годъ.	Вскрытіе.	Замерзаніе.	Свободн. отъ льда.
1799	IV 17	XII 23	250	1834	III 10	XII 27	292
1800	IV 12	XII 19	251	1835	III 4	XI 13	254
1801	III 19	I 15 1802	302	1836	III 14	XII 30	291
1802	III 19	XII 26	282	1837	III 28	XII 12	259
1803	IV 7	XII 2	239	1838	IV 4	XII 1	241
1804	IV 3	XI 21	232	1839	IV 14	XII 5	235
1805	IV 23	XI 25	216	1840	IV 9	XII 13	248
1806	III 22	I 10 1807	294	1841	IV 12	XII 23	255
1807	III 9	I 17 1808	314	1842	III 31	XII 25	269
1808	IV 21	XII 3	226	1843	II 7	I 12 1844	339
1809	IV 10	XI 25	229	1844	IV 9	XII 4	239
1810	III 11	XI 27	261	1845	IV 21	—	—
1811	III 12	XII 25	288	1856	IV 7	XII 23	260
1812	IV 1	XII 9	252	1857	III 31	I 3 1858	278
1813	III 24	XII 31	282	1858	IV 5	XI 17	226
1814	IV 6	XII 30	268	1859	III 15	XII 6	266
1815	IV 3	XII 11	252	1860	IV 4	XII 4	244
1816	III 18	XII 12	269	1861	III 27	XII 10	258
1817	III 4	I 3 1818	305	1862	III 26	XI 19	238
1818	III 4	XII 20	291	1863	III 5	XII 7	277
1819	III 28	XII 8	255	1864	III 11	XII 1	265
1820	IV 9	XII 21	256	1865	IV 9	XII 15	250
1821	III 22	I 29 1822	313	1866	III 17	XII 1	259
1822	II 27	XII 21	297	1867	IV 6	XI 29	237
1823	III 22	I 15 1824	299	1868	IV 6	XI 22	230
1824	III 10	I 29 1825	325	1869	III 9	II 3 1870	331
1825	IV 17	I 3 1826	261	1870	IV 6	XII 26	264
1826	III 29	I 26 1827	303	1871	IV 4	XII 26	266
1827	III 10	XII 13	278	1872	III 28	XII 31	278
1828	III 27	XII 23	271	1873	III 19	I 10 1874	297
1829	IV 9	XI 28	233	1874	III 26	I 6 1875	286
1830	IV 5	I 11 1831	281	1875	IV 9	XII 7	242
1831	IV 9	XII 6	241	1876	III 23	XI 25	247
1832	IV 3	XI 26	237	1877	III 20	XII 23	278
1833	III 30	I 7 1834	283	1878	III 30	XII 20	265

Fig. S1. Example of a transcribed data source (Dnjepr, Kyiv), from Rykachev.[11] The columns indicate freezing date, thawing date, and the duration of ice-free conditions.

Table S1. Phenological series used, coordinates, proxy name, years covered, season, station used for calibration, calibration years, calibration months, correlation coefficient in the calibration, and reference (* = stations already assimilated into EKF400v2, V = used only for evaluation, °x = x outliers removed, T = transformed, E = excluded due to low skill). BEST = closest grid point in Berkeley Earth surface temperature data set.

#	Pheno series	Location	Lat	Lon	Proxy	Years	Season	Station	Cal.yrs	Cal.mons	r	Reference
1V	Amur River	Nikolayevsk	53.13	140.72	freezing date	1855-1908	fall	Nikolayevsk 53.15N, 140.70E	1855-1908	Oct-Nov	0.58	Shostakovich 1909[1]
2V	Amur River	Nikolayevsk	53.13	140.72	thawing date	1855-1907	spring	Nikolayevsk 53.15N, 140.70E	1855-1907	Mar-May	0.71	Shostakovich 1909[1]
3	Angara River	Irkutsk	52.3	106.33	freezing date	1721-1956	fall	Irkutsk 52.27N, 104.32E	1851-1950	Oct-Jan	0.65	Shostakovich 1909[1] Magnuson et al. 2000[2]
4V	Sprig pheno	Beijing	39.90	116.40	phenology (various)	1741-1832	spring	BEST plus Dove (adjusted to 1851-1900 average), *n*=19	1757-1832	Average Feb-Apr	0.28	Liu & Fang 2017[3]
5	First snowfall	Annecy	45.90	6.13	freezing date	1776-1911	fall	Lyon 45.70N, 4.70E	1852-1950	Oct-Nov	0.54	Mougin 1912[4]
6	Danube	Budapest	47.49	19.03	freezing date	1783-2002	fall	Budapest 47.52N, 19.03E	1851-1950	Nov-Dec	0.56	Takács et al. 2018[5]
7	Danube	Budapest	47.49	19.03	thawing date	1775-2000	spring	Budapest 47.52N, 19.03E	1851-1950	Jan-Mar	0.81	Takács et al. 2018[5]
8T	Cherry tree	Swiss Plateau	47.25	8.00	flowering date	1721-2003	spring	Zürich 47.38N, 8.57E	1864-1950	Jan-May	0.73	Rutishauser 2003[6]
9*V	Cherry tree	Kyoto	35.00	135.67	flowering date	1600-2003	spring	Kyoto 35.02N, 135.73E	1881-1950	Mar-Apr	0.70	Aono & Kazui 2008[7] Aono & Saito 2010[8]
10*V	Cherry tree	Tokyo	35.68	139.77	flowering date	1636-1905	spring	Tokyo 35.68N, 139.77E	1877-1905	Mar	0.85	Aono 2015[9]
11	Connecticut River	Middletown	41.56	72.65	freezing date	1780-1840	fall	New Haven 41.30N, -72.90E	1781-1840	Dec	0.66	Barrat 1840[10]
12°5	Connecticut River	Middletown	41.56	72.65	thawing date	1762-1840	spring	New Haven 41.30N, -72.90E	1781-1840	Jan-Feb	0.51	Barrat 1840[10]
13	Dnieper River	Dnipropetrovsk	48.45	35.07	freezing date	1819-1880	fall	Poltava 49.60N, 34.55E	1824-1866	Dec	0.78	Rykachev 1886[11]
14	Dnieper River	Dnipropetrovsk	48.45	35.07	thawing date	1802-1879	spring	Poltava 49.60N, 34.55E	1824-1866	Feb-Mar	0.67	Rykachev 1886[11]
15	Dnieper River	Kyiv	50.45	30.5	freezing date	1800-1879	fall	Kyiv 50.40N, 30.57E	1812-1879	Nov-Dec	0.78	Rykachev 1886[11]
16	Dnieper River	Kyiv	50.45	30.5	thawing date	1799-1878	spring	Kyiv 50.40N, 30.57E	1812-1878	Feb-Mar	0.82	Rykachev 1886[11]
17T	Albany River	Fort Albany	52.23	-81.60	thawing date	1722-1939	spring	Moosone 51.27N, -80.65E	1878-1940	Apr-May	0.76	Magne 1981[12] Catchpole et al. 1976[13]
18	Haarlem-Leiden Channel	Haarlem	52.41	4.65	days frozen	1634-1839	winter	De Bilt 52.10N, 5.18E	1707-1757	Jan-Mar	0.81	de Vries 1978[14]
19	Sea ice	Iceland	64.96	-19.02	sea ice severity	1601-2000	year	Stykkisholmur 65.08N, -22.73E	1851-1950	Dec-May	0.64	Ogilvie 1984[15] Ogilvie & Jónsdóttir 2000[16]
20	Irtysh River	Tobolsk	58.2	68.23	freezing date	1813-1903	fall	Tobolsk 58.15N, 68.25E	1832-1903	Oct-Nov	0.67	Rykachev 1886[11] Shostakovich 1909[1]
21	Irtysh River	Tobolsk	58.2	68.23	thawing date	1812-1902	spring	Tobolsk 58.15N, 68.25E	1832-1902	Apr-May	0.60	Rykachev 1886[11] Shostakovich 1909[1]
22V	Spring melt	Churchill	58.77	94.17	thawing date	1719-1838	spring	BEST	1775-1849	Mar-Apr	0.52	Catchpole et al. 1976[13]

23V	Kennebec River	Gardiner	44.18	-69.77	thawing date	1785-1857	spring	Boston 42.37N, -71.03E	1791-1840	Feb-Mar	0.57	Gardiner 1858[17]
24	Lake Champlain	Burlington	44.48	-73.21	freezing date	1816-2001	fall	Burlington 44.47N, -73.15E	1893-1950	Jan	0.64	Benson et al. 2000/2020[18]
25T	Lake Oneida	Brewerton	43.24	-76.14	thawing date	1827-2003	spring	Oswego 43.46N, -76.49E	1890-1950	Mar	0.73	Benson et al. 2000/2020[18]
26	Lake Sebago	Standish	43.87	-70.57	thawing date	1807-2003	spring	Portland 43.65N, -70.30E	1886-1950	Jan-Apr	0.80	Hodgkins 2010[19] Benson et al. 2000/2020[18]
27	Lake Suwa	Suwa	36.04	138.08	freezing date	1600-1954	fall	Tokyo 35.68N, 139.77E	1877-1950	Nov-Dec	0.59	Arakawa 1954[20] Benson et al. 2000/2020[18]
28	Lena River	Yakutsk	62.02	129.72	freezing date	1827-1908	fall	Yakutsk 62.02N, 129.72E	1830-1907	Oct	0.52	Shostakovich 1909[1]
29T	Lena River	Yakutsk	62.02	129.72	thawing date	1827-1906	spring	Yakutsk 62.02N, 129.72E	1830-1907	May-May	0.54	Shostakovich 1909[1]
30T	Lake Mälaren	Västerås	59.61	16.55	thawing date	1712-1999	spring	Stockholm 59.33N, 18.05E	1851-1950	Jan-May	0.85	Hildebrandsson 1905[21] Eklund 1999[22]
31*V	Maple tree	Kyoto	35.00	135.67	leaf coloring	1600-2003	fall	Kyoto 35.02N, 135.73E	1882-1950	Oct-Nov	0.53	Aono & Tani 2014[23]
32	Oak tree	Norwich	52.63	1.29	leaving date	1746-1947	spring	Cambridge 52.20N, 0.10E	1872-1947	Jan-Apr	0.79	Sparks & Carey 1995[24]
33	Miramichi River	New Brunswick	46.12	-64.70	thawing date	1830-1956	spring	Moncton, 46.12N, -64.68E	1901-1950	Mar-Apr	0.81	Benson et al. 2000/2020[18]
34	Mississippi River	Davenport	41.52	-90.58	freezing date	1842-1935	fall	Davenport 41.62N, -90.58E	1873-1935	Nov-Dec	0.68	Shipman 1937[25]
35	Mississippi River	Davenport	41.52	-90.58	thawing date	1842-1935	spring	Davenport 41.62N, -90.58E	1873-1935	Nov-Mar	0.79	Shipman 1937[25]
36°1	Newa River	St. Petersburg	59.93	30.35	freezing date	1707-1883	fall	St. Petersburg 59.97N, 30.30E	1751-1850	Nov	0.62	Rykachev 1886[11]
37	Newa River	St. Petersburg	59.93	30.35	thawing date	1706-1882	spring	St. Petersburg 59.97N, 30.30E	1751-1850	Mar-Apr	0.79	Rykachev 1886[11]
38	Sea ice	Newfoundland	49.66	54.00	sea ice area	1810-2003	spring	St. John's 47.62N, -52.73	1851-1950	Jan-Mar	0.62	Hill & Jones 1990[26]
39	Northern Dvina River	Arkhangelsk	64.53	40.52	freezing date	1735-1880	fall	Arkhangelsk 64.50N, 40.73E	1814-1880	Oct-Dec	0.56	Rykachev 1886[11]
40	Northern Dvina River	Arkhangelsk	64.53	40.52	thawing date	1734-1879	spring	Arkhangelsk 64.50N, 40.73E	1814-1879	Apr-May	0.64	Rykachev 1886[11]
41	Ob River	Barnaul	53.33	83.80	freezing date	1752-1906	fall	Barnaul 53.43N, 83.52E	1851-1906	Oct-Nov	0.68	Rykachev 1886[11], Shostakovich 1909[1]
42	Ob River	Barnaul	53.33	83.80	thawing date	1751-1905	spring	Barnaul 53.43N, 83.52E	1851-1905	Mar-Apr	0.84	Rykachev 1886[11], Shostakovich 1909[1]
43	Onega River	Onega	63.90	38.13	freezing date	1781-1879	fall	Arkhangelsk 64.50N, 40.73E	1814-1879	Nov	0.71	Rykachev 1886[11]
44	Onega River	Onega	63.90	38.13	thawing date	1780-1878	spring	Arkhangelsk 64.50N, 40.73E	1814-1878	Apr-May	0.73	Rykachev 1886[11]
45	Pond	Karpinsk	59.75	60.00	freezing date	1840-1893	fall	Bogoslowsk 59.80N, 60.10E	1839-1880	Oct-Nov	0.72	Shostakovich 1909[1]
46	Pond	Karpinsk	59.75	60.00	thawing date	1839-1894	spring	Bogoslowsk 59.80N, 60.10E	1839-1880	May-May	0.49	Shostakovich 1909[1]
47	Randsfjorden	Gran	60.31	10.43	thawing date	1758-2003	spring	Oslo Blindern 59.90N, 10.70E	1851-1950	Mar-Apr	0.71	Nordli et al. 2007[27]
48	Red River	Winnipeg	50.00	-97.25	freezing date	1800-1982	fall	Winnipeg 49.90N, -97.23E	1873-1950	Nov	0.54	Rannie 1983[28]
49	Red River	Winnipeg	50.00	-97.25	thawing date	1800-1994	spring	Winnipeg 49.90N, -97.23E	1873-1950	Mar-Apr	0.82	Rannie 1983[28]
50V	Moose River	Moose Factory	51.26	-80.59	thawing date	1736-1864	spring	BEST	1820-1871	Apr	0.66	Catchpole et al. 1976[13]
51	Syssola River	Syktyvkar	61.67	50.85	thawing date	1773-1879	spring	Syktyvkar 61.72N, 50.83E	1818-1868	Feb-Apr	0.51	Rykachev 1886[11]
52	Tallinn Port	Tallinn	59.40	24.75	thawing date	1600-1996	spring	Tallinn 59.42N, 24.80E	1851-1950	Jan-Apr	0.77	Tarand & Nordli, 2001[29]

53[T*V]	Tornio River	Tornio	65.85	24.16	thawing date	1693-2003	spring	Haparanda 65.83N, 24.15E	1860-1950	Apr-May	0.85	Kuusisto & Elo 2000[30] Magnuson et al. 2000[2]
54	Toronto Harbour	Toronto	43.63	-79.4	freezing date	1823-1987	fall	Toronto 43.67N, -79.4E	1823-1919	Nov-Dec	0.55	Benson et al. 2000/2020[18]
55	Toronto Harbour	Toronto	43.63	-79.4	thawing date	1823-1986	spring	Toronto 43.67N, -79.4E	1823-1919	Feb-Mar	0.62	Benson et al. 2000/2020[18]
56	Vaga River	Velsk	61.08	42.12	thawing date	1807-1878	spring	Arkhangelsk 64.50N, 40.73E	1814-1878	Apr-May	0.67	Rykachev 1886[11]
57	Vistula River	Warshaw	51.27	21.03	freezing date	1725-1880	fall	Warszaw 52.17N, 20.97E	1780-1879	Nov-Dec	0.68	Rykachev 1886[11]
58	Vistula	Warshaw	51.27	21.03	thawing date	1725-1879	spring	Warszaw 52.17N, 20.97E	1780-1879	Feb	0.51	Rykachev 1886[11]
59	Volga River	Astrakhan	46.33	48.05	freezing date	1805-1880	fall	Astrakhan 46.28N, 48.05E	1837-1880	Dec	0.72	Rykachev 1886[11]
60	Volga River	Astrakhan	46.33	48.05	thawing date	1804-1879	spring	Astrakhan 46.28N, 48.05E	1837-1879	Jan-Apr	0.87	Rykachev 1886[11]
61	Daugava River	Riga	56.95	24.1	freezing date	1602-1882	fall	Riga 56.97N, 24.05E	1795-1882	Oct-Nov	0.64	Rykachev 1886[11]
62	Daugava River	Riga	56.95	24.1	thawing date	1601-1881	spring	Riga 56.97N, 24.05E	1795-1881	Jan-Apr	0.76	Rykachev 1886[11]
63	Yenisei River	Yeniseysk	58.45	92.13	freezing date	1822-1908	fall	Yeniseysk 58.45N, 92.15E	1872-1908	Oct-Nov	0.84	Rykachev 1886[11], Shostakovich 1909[1]
64	Yenisei River	Yeniseysk	58.45	92.13	thawing date	1790-1907	spring	Yeniseysk 58.45N, 92.15E	1872-1907	Jan-Apr	0.63	Rykachev 1886[11], Shostakovich 1909[1]
65	Ufa River	Ufa	54.72	55.95	freezing date	1781-1880	fall	Kazan 55.60N, 49.28E	1812-1879	Oct	0.45	Rykachev 1886[11]
66	Ufa River	Ufa	54.72	55.95	thawing date	1813-1879	spring	Kazan 55.60N, 49.28E	1812-1879	Apr	0.51	Rykachev 1886[11]
67	Severn River	Fort Severn	55.99	-87.63	freezing date	1761-1898	fall	York Factory 57.00N, -92.30E	1775-1849	Nov	0.51	Magne 1981[12]
68	Severn River	Fort Severn	55.99	-87.63	thawing date	1763-1897	spring	York Factory 57.00N, -92.30E	1775-1849	Apr	0.59	Magne 1981[12]
69	Hudson River	Albany	42.65	73.76	freezing date	1790-1856	fall	New Haven 41.30N, -72.90E	1790-1856	Nov-Dec	0.73	NY Almanach 1857[31]
70	Hudson River	Albany	42.65	73.76	thawing date	1790-1856	spring	New Haven 41.30N, -72.90E	1790-1856	Feb-Mar	0.74	NY Almanach 1857[31]
71	Kama River	Perm	58.02	56.27	freezing date	1792-1880	fall	Sverdlovsk 56.80N, 60.60E	1832-1880	Nov	0.51	Rykachev 1886[11]
72	Kama River	Perm	58.02	56.27	thawing date	1767-1879	spring	Sverdlovsk 56.80N, 60.60E	1832-1879	Apr	0.70	Rykachev 1886[11]
73[V]	Lena River	Kirensk	57.78	108.05	freezing date	1816-1855	fall	Irkutsk 52.27N, 104.32E	1816-1855	Oct	0.62	Rykachev 1886[11]
74[V]	Oulujoki	Oulu	65.02	25.50	thawing date	1784-1880	spring	Helsinki, 60.3N, 25.0E	1830-1880	Feb-May	0.56	Rykachev 1886[11]
75	Sukhona River	Veliky Ustyug	60.77	46.30	freezing date	1762-1880	fall	Yakutsk 62.02N, 129.72E	1818-1868	Oct-Dec	0.45	Shostakovich 1909[1]
76	Sukhona River	Veliky Ustyug	60.77	46.30	thawing date	1761-1879	spring	Yakutsk 62.02N, 129.72E	1818-1868	Feb-Apr	0.79	Shostakovich 1909[1]
77	Vologda River	Vologda	59.23	39.88	thawing date	1781-1878	spring	Moskva 55.83N, 37.62E	1781-1857	Apr	0.77	Rykachev 1886[11]
78[V]	Aura River	Turku	60.75	22.45	thawing date	1740-1839	spring	Stockholm, 59.33N, 18.05E	1800-1839	Mar-Apr	0.86	Rykachev 1886[11]
79	Vyatka River	Vyatka	58.77	49.70	thawing date	1800-1878	spring	Syktyvkar 61.72N, 50.83E	1812-1876	Apr	0.70	Rykachev 1886[11]
80[V]	Lake St Moritz	St. Moritz	46.49	9.85	thawing date	1832-2012	spring	Segl-Maria 46.43N, 9.76E	1864-1950	Apr-May	0.67	Livingstone 1997[32]
81	Horse chestnut	Geneva	46.20	6.14	flowering date	1808-2020	spring	Geneva 46.25N, 6.13E	1864-1950	Feb-Mar	0.72	Defila & Clot 2001[33]
82[°1]	Grapes	Klosterneuburg	48.30	16.33	flowering date	1732-1878	spring	Vienna 48,25N, 16.36E	1776-1878	Apr-May	0.73	Maurer 2009[34]

Table S2. Reconstructions used in the paper: Method used, prior and number of years in analog pool, number of observations assimilated, and abbreviation

Method	Prior	Obs.	Abbreviation
Bayesian Reweighting	CCC400, all 8940 years	68	BRW_{CCC}
Bayesian Reweighting	NCAR-LME, all 13000 years	68	BRW_{NCAR}
Bayesian Reweighting	EKF400v2, 30 members of corresponding year	68	BRW_{EKF}
Bayesian Reweighting	CCC400, all 8940 years	82	$XBRW_{CCC}$
Bayesian Reweighting	NCAR-LME, all 13000years	82	$XBRW_{NCAR}$
Closest Analog	CCC400, all 8940 years	68	BRW_{CCC}
Closest Analog	NCAR-LME, all 13000years	68	BRW_{NCAR}

Table S3. Pearson correlation coefficients between the reconstruction (BRW_{CCC}, BRW_{NCAR}, BRW_{EKF}, $XBRW_{CCC}$, and $XBRW_{NCAR}$) and the temperature data sets BEST, GISSTEMP, and EKF400v2 for different periods.

Correlation coefficients	BRW_{CCC}	BRW_{NCAR}	BRW_{EKF}	$XBRW_{CCC}$	$XBRW_{NCAR}$
BEST (1881-1905)	0.687	0.543	0.927	0.699	0.588
BEST (1751-1905)	0.563	0.530	0.667	0.576	0.496
CRUTEMP5 (1881-1905)	0.540	0.585	0.758	0.609	0.674
GISTEMP4 (1881-1905)	0.558	0.567	0.806	0.609	0.615
EKF400v2 (1881-1905)	0.564	0.500	0.999	0.564	0.531
EKF400v2 (1701-1905)	0.569	0.498	0.947	0.582	0.453

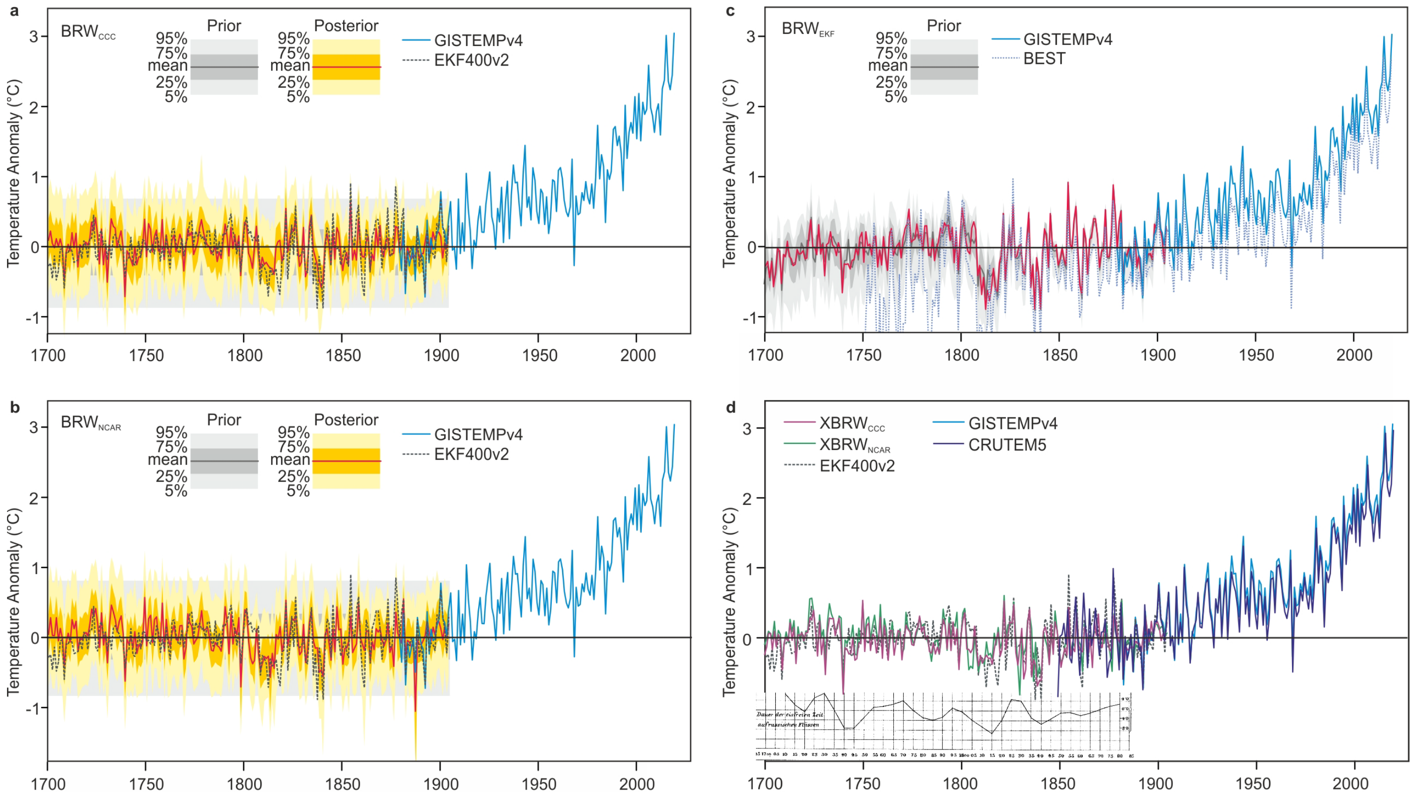

Fig. S2. Cold-season temperatures from 1700 to present. Time series of cold-season temperatures over land areas between 35 and 70°N from **a** BRW$_{CCC}$, **b** BRW$_{NCAR}$, **c** BRW$_{EKF}$ and **d** XBRW$_{CCC}$ as well as corresponding time series from various instrumental data sets. Curves are expressed as anomalies with respect to 1881-1905. Average anomaly of ice-free days in Russian rivers from Brückner,[35] given in 5-yr averages, is included in the lower part of panel **d**.

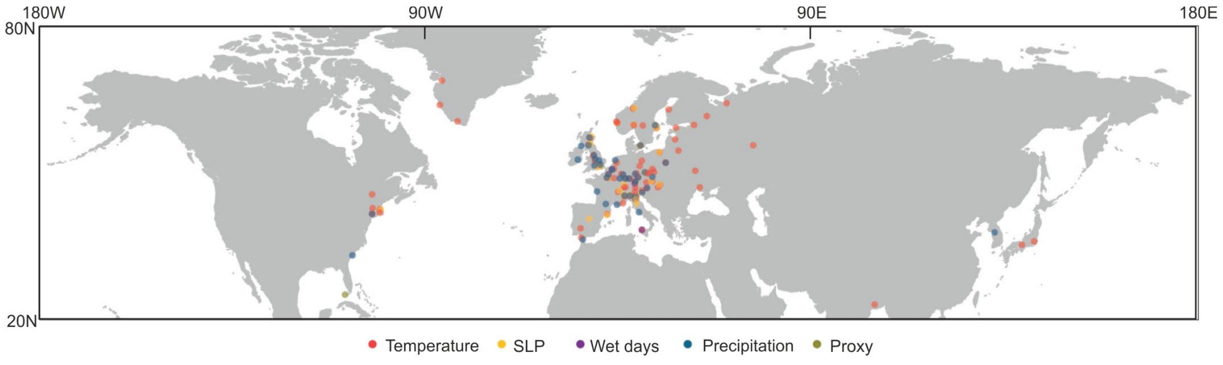

Fig. S3. Data coverage in EKF400v2. Data assimilated into EKF400v2 during the cold season (Oct-Mar) 1808/9 to 1815/16 (temp2 = 2 m temperature, slp = sea-level pressure, wetdays = number of wet days, prcip = precipitation, prox = proxies).

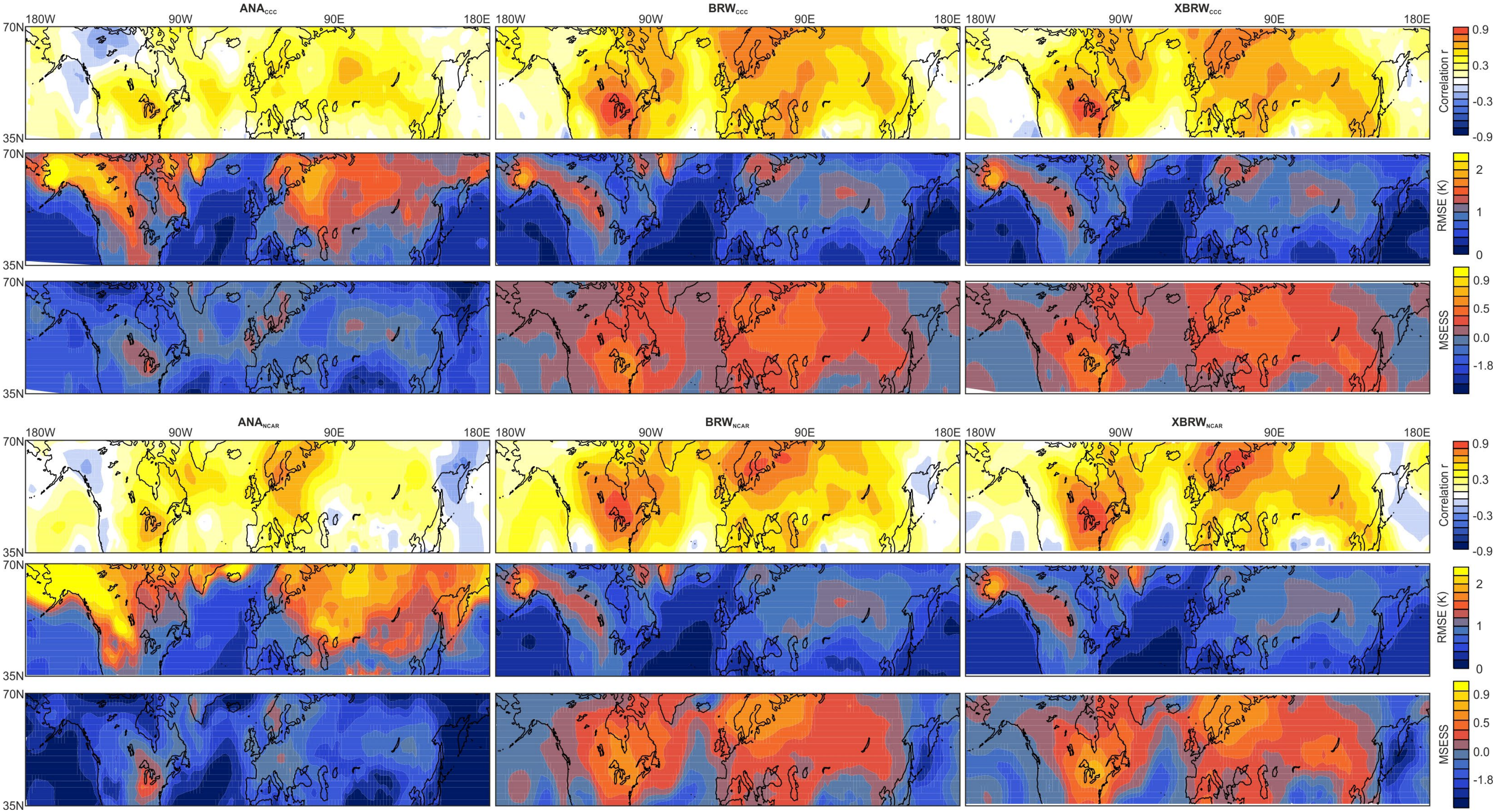

Fig. S4. Evaluation of temperature field reconstructions. ANA_{CCC}, BRW_{CCC} and $XBRW_{CCC}$ as well as ANA_{NCAR}, BRW_{NCAR} and $XBRW_{NCAR}$ are evaluated against EKF400v2, 1851-1900, using the Pearson correlation coefficient (top), the root mean squared error (second row) and the mean squared error skill score (third row). Note that $XBRW_{NCAR}$ and $XBRW_{CCC}$ are not independent of EKF400v2, four series overlap.

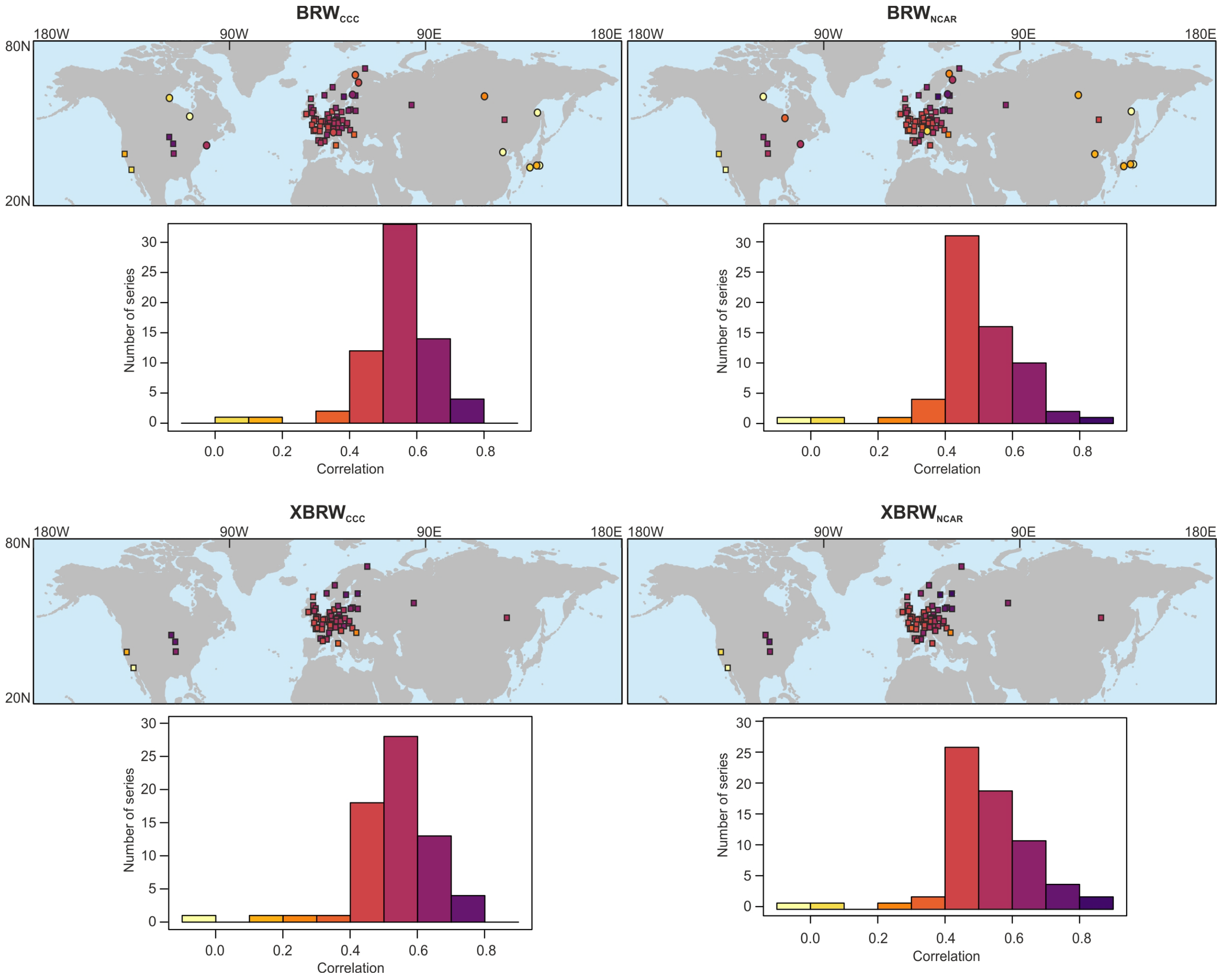

Fig. S5. Independent evaluation of the reconstructions. Correlation between cold-season reconstructions and GHCN station data (squares, n = 67) in the period 1851-1900. Also shown are correlations between independent documentary data and the same data forward modeled in the reconstructions (circles in top row, over the longest period of record possible). The lower figures show histograms of the correlations with GHCN stations.

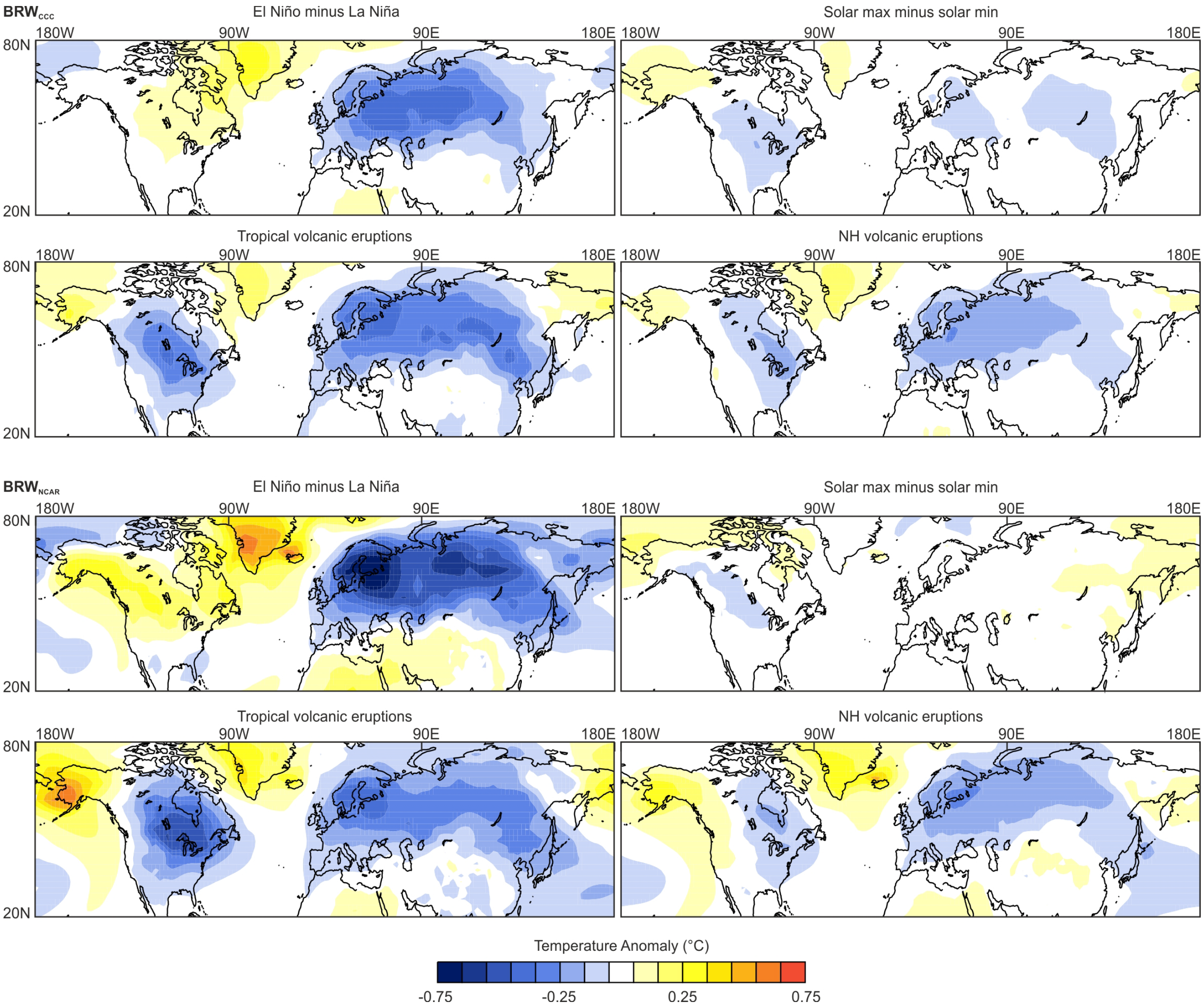

Fig. S6. Effect of ENSO, solar and volcanic forcing on cold season temperatures. Composite temperature differences of El Niño minus La Niña, solar maximum minus solar minimum, tropical and northern hemispheric volcanic eruptions (both against 1851-1900).

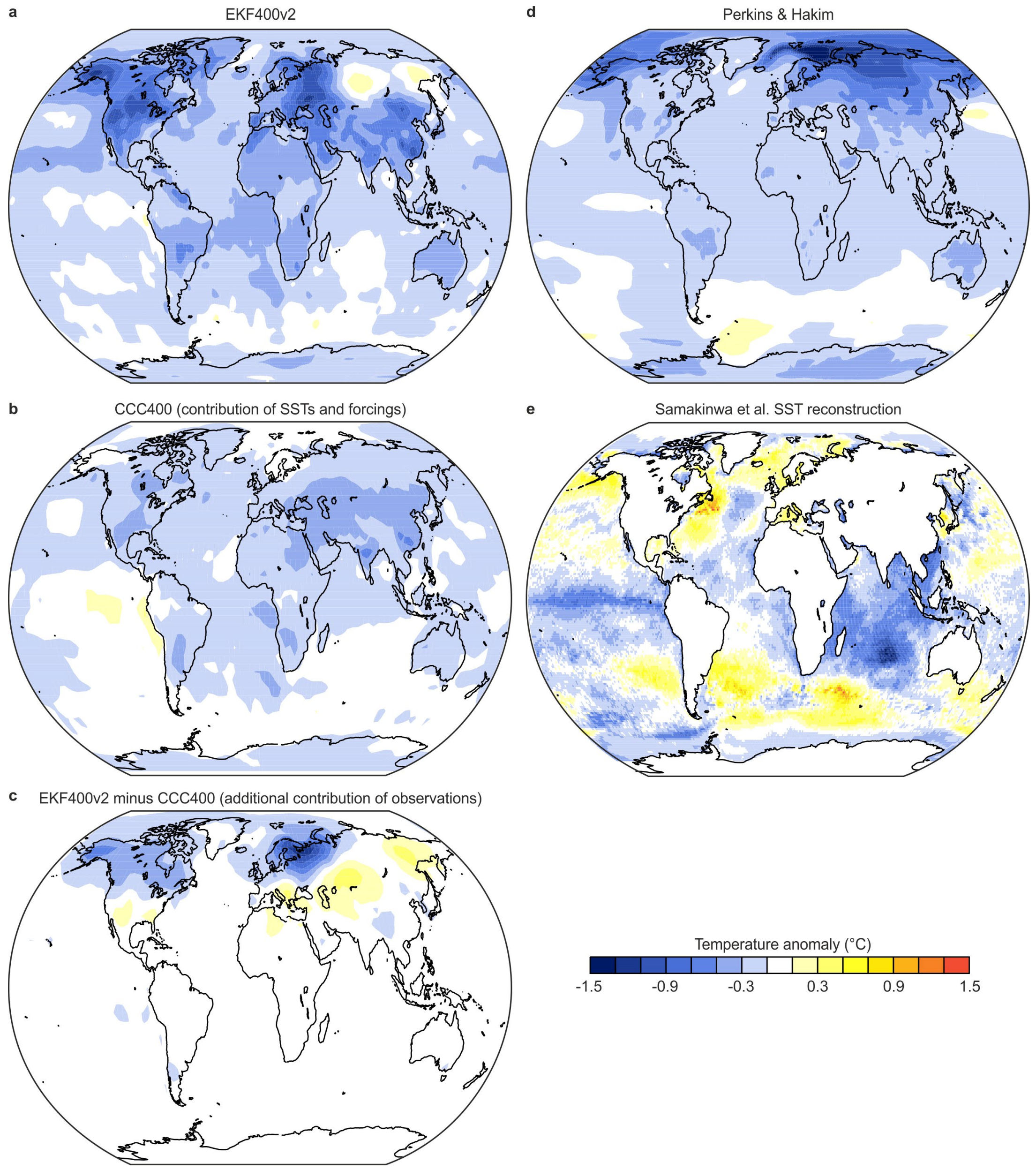

Fig. S7. Averaged anomalies (relative to 1851-1900) from 1808/9 to 1815/6. in **a,** EKF400v2, **b,** CCC400 model simulations. **c,** difference between EKF400v2 and CCC400, **d** reconstructions by Perkins & Hakim (annual mean, 1809-1816) and **e** Samakinwa et al. sea-surface temperature reconstructions.

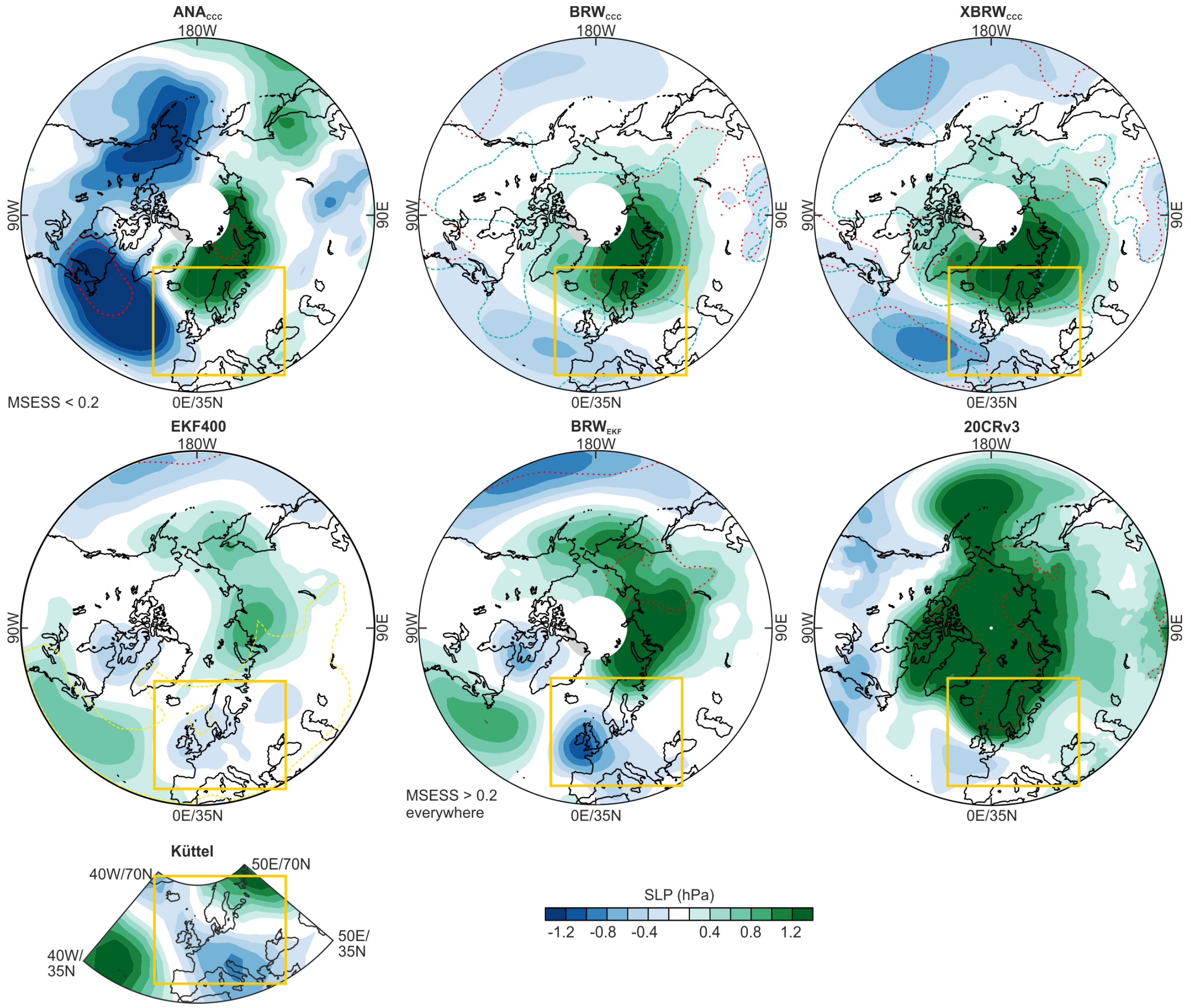

Fig. S8. Sea-level pressure anomalies (relative to 1851-1900) during 1808/9 to 1815/16 in seven different SLP data sets. Dashed red lines show anomalies outside 1 standard deviation of the 1851-1900 period. Green dashed lines indicate MSESS = 0.2 in our reconstructions. The yellow dashed lines indicate MSESS = 0.2 in EKF400.

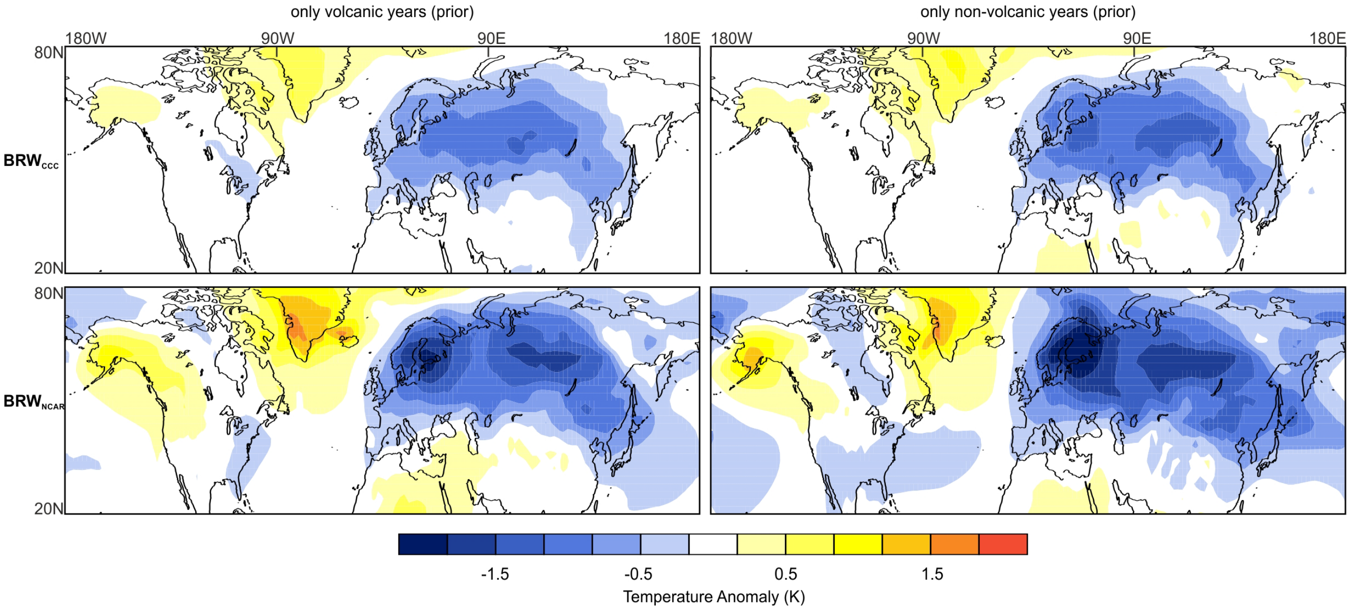

Fig. S9. Temperature anomalies during the two volcanically perturbed winter 1809/10 and 1815/16 in two reconstructions that used either only volcanic or only non-volcanic years in the prior.

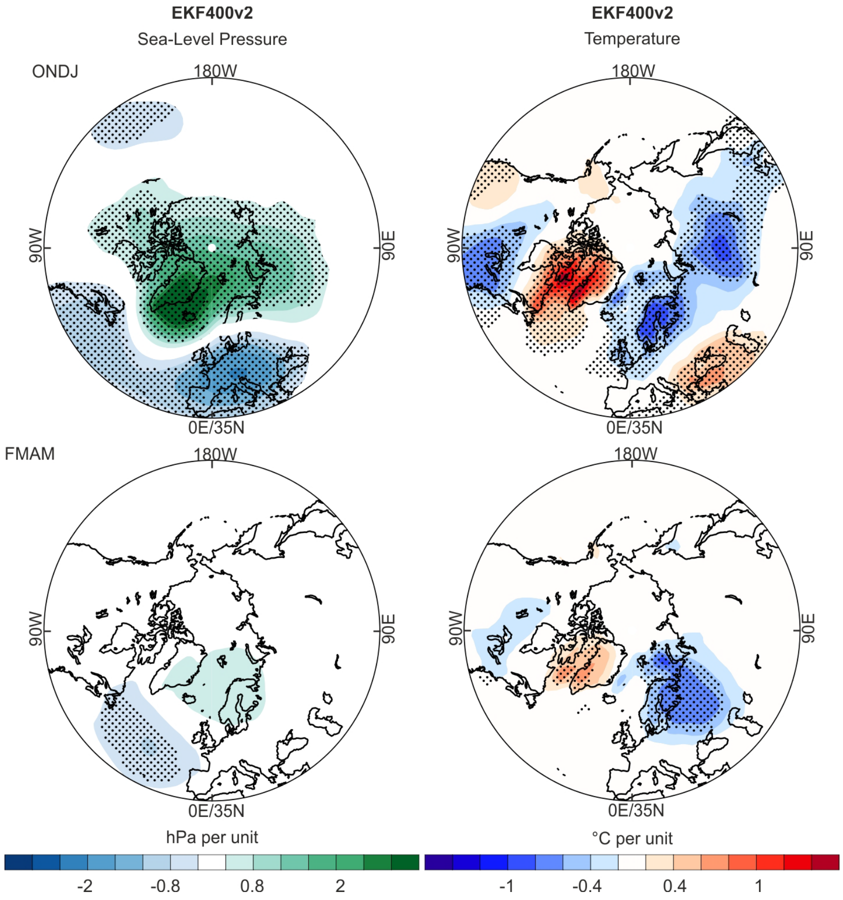

Fig. S10. Same as Fig. 4, but using EKF400v2 (snow cover is not available for this data set).

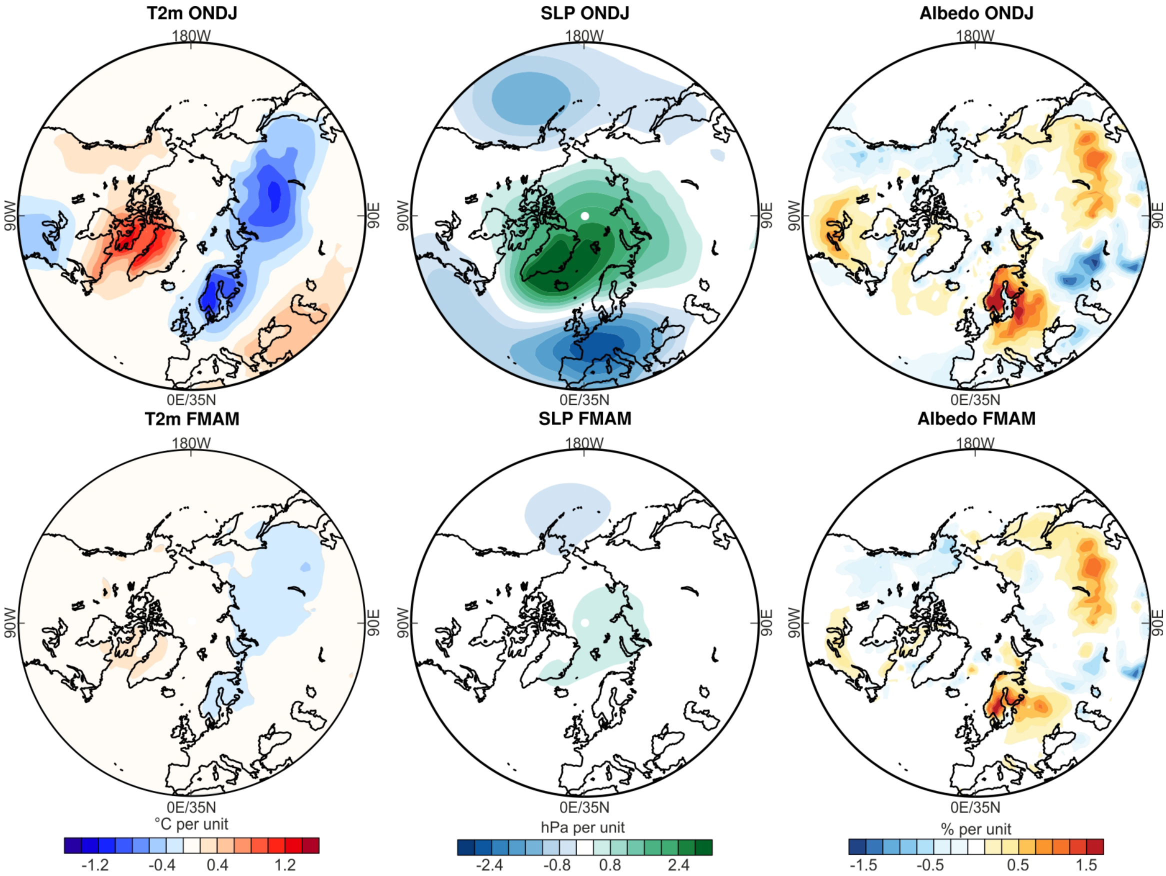

Fig. S11. Same as Fig. 4 but using the ECHAM6 simulations (using albedo rather than snow cover).

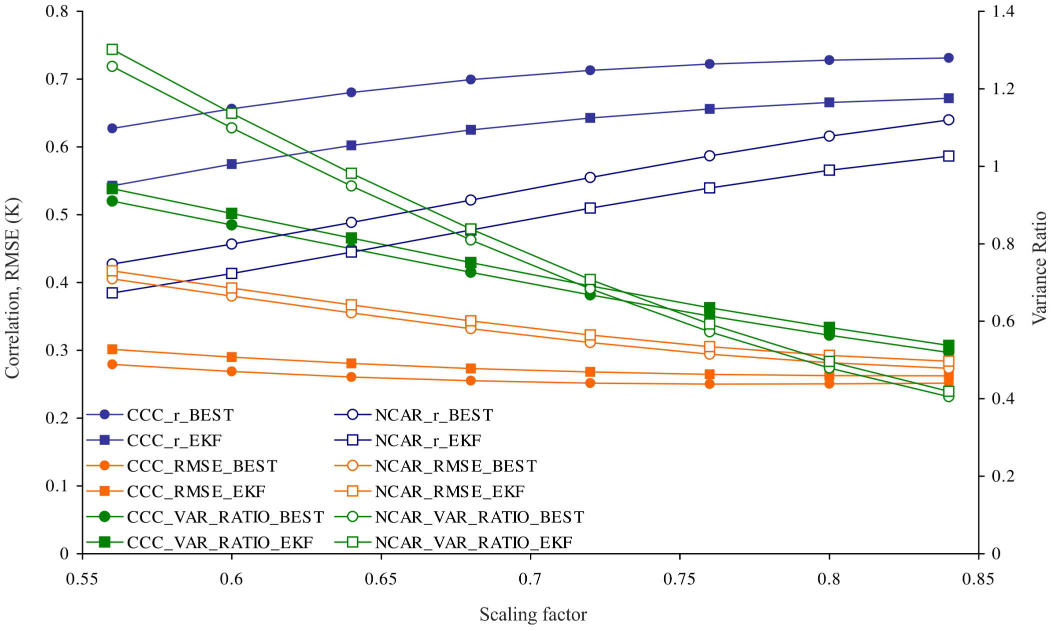

Fig. S12. Choice of scaling factor. Correlation, RMSE and variance ratio (defined as the variance in the reconstruction divided by that variance in the reference data set) for the cold-season mean temperature averaged over the northern extratropical as a function of the scaling factor used when converting the distance measure to a weight. Shown are results for BRW_{CCC} (filled symbols) and BRW_{NCAR} (open symbols) for two reference data sets, namely EKF400 and BEST. The chosen scaling factor exhibits high correlations and low RMSE and retains half of the variance. The reference period in all cases is 1851-1900.

Supplementary references

1. Shostakovich, V. B. *Opening and freezing of waters of Asian Russia*. (паровая типо-литография П. И. Макушина и В. М. Посохина, 1909). [in Russian]
2. Magnuson, J. J. *et al.* Historical trends in lake and river ice cover in the Northern Hemisphere. *Science* **289**, 1743–1746 (2000).
3. Liu, Y. & Fang, X. Reconstruction of spring phenology and temperature in Beijing, China, from A.D. 1741 to 1832. *Int. J. Climatol.* **37**, 5080–5088 (2017).
4. Mougin, M. Etudes glaciologiques en Savoie. in *Études Glaciologiques*: Volume 3 (eds. Mougin, P. & Gaurier, L.) 1–113 (Imprimerie nationale, 1912).
5. Takács, K., Kern, Z. & Pásztor, L. Long-term ice phenology records from eastern–central Europe. *Earth System Science Data* **10**, 391–404 (2018).
6. Rutishauser, T. Cherry Tree Phenology. Interdisciplinary Analyses of Phenological Observations of the Cherry Tree in the Extended Swiss Plateau Region and Their Relation to Climate Change. (University of Bern, Bern, 2003).
7. Aono, Y. & Kazui, K. Phenological data series of cherry tree flowering in Kyoto, Japan, and its application to reconstruction of springtime temperatures since the 9th century. *Int. J. Climatol.* **28**, 905–914 (2008).
8. Aono, Y. & Saito, S. Clarifying springtime temperature reconstructions of the medieval period by gap-filling the cherry blossom phenological data series at Kyoto, Japan. *Int. J. Biometeorol.* **54**, 211–219 (2010).
9. Aono, Y. Cherry blossom phenological data since the seventeenth century for Edo (Tokyo), Japan, and their application to estimation of March temperatures. *Int. J. Biometeorol.* **59**, 427–434 (2015).
10. Barrat, J. M. D. First Appearance of Ice, the Closing and the time of Opening of the Conneticut River at Middletown. *The American journal of science and arts.* **39**, 88–90 (1840).
11. Rykachev, M. *Openings and freezings of rivers in the Russian Empire*. (тип. Имп. Акад. наук, 1886). [in Russian]
12. Magne, M. A. Two Centuries of River Ice Dates in Hudson Bay Region from Historical Sources. (University of Manitoba, Winnipeg, 1981).
13. Catchpole, A. J. W., Moodie, D. W. & Milton, D. Freeze-Up and Break-Up of Estuaries on Hudson Bay in the Eighteenth and Nineteenth Centuries. *The Canadian Geographer/Le Géographe canadien* **20**, 279–297 (1976).
14. de Vries, J. Histoire du climat et économie: des faits nouveaux, une interprétation différente. *Annales. Histoire, Sciences Sociales* **32**, 198–226 (1977).
15. Ogilvie, A. E. J. The past climate and sea-ice record from Iceland, Part 1: Data to A.D. 1780. *Climatic Change* **6**, 131–152 (1984).

16. Ogilvie, A. E. J. & Jónsdóttir, I. Sea Ice, Climate, and Icelandic Fisheries in the Eighteenth and Nineteenth Centuries. *ARCTIC* **53**, 383–394 (2000).
17. Gardiner, R. H. Obervations on the Opening and Closing of Kennebec River, Maine. *Annual report of the Board of Regents of the Smithsonian Institution* 434–436 (1858).
18. Benson, B., J. Magnuson, and S. Sharma. 2000, updated 2020. Global Lake and River Ice Phenology Database, Version 1. Boulder, Colorado USA. NSIDC: National Snow and Ice Data Center. doi:https://doi.org/10.7265/N5W66HP8. [11 Dec 2020].
19. Hodgkins, G. A. *Historical Ice-Out Dates for 29 Lakes in New England. U.S. Geological Survey-Open-File Report 2010–1214* (2010).
20. Arakawa, H. Fujiwhara on five centuries of freezing dates of Lake Suwa in the Central Japan. *Archiv für Meteorologie, Geophysik und Bioklimatologie Serie B* **6**, 152–166 (1954).
21. Hildebrandsson, H. H. Sur le prétendu changement du climat européen en temps historiques. *Nova acta Regiae Societatis Scientiarum Upsaliensis* **4**, 5 (1905).
22. Eklund, A. Isläggning och islossning i svenska sjöar. (Long observation series of ice freeze and break up dates in swedish lakes). *SMHI Hydrologi* **81**, 1–24 (1999). [in Swedish]
23. Aono, Y. & Tani, A. Autumn temperature deduced from historical records of autumn tints phenology of maple tree in Kyoto, Japan. *Climate in Biosphere* **14**, 18–28 (2014).
24. Sparks, T. H. & Carey, P. D. The Responses of Species to Climate Over Two Centuries: An Analysis of the Marsham Phenological Record, 1736-1947. *Source: Journal of Ecology* **83**, 321–329 (1995).
25. Shipman, T. G. Ice-conditions on the Mississippi River at Davenport, Iowa. *Northwest Science* **12**, 590–594 (1938).
26. Hill, B. T. & Jones, S. J. The Newfoundland ice extent and the solar cycle from 1860 to 1988. *J. Geophys. Res.* **95**, 5385 (1990).
27. Nordli, Ø., Lundstad, E. & Ogilvie, A. E. J. A late-winter to early-spring temperature reconstruction for southeastern Norway from 1758 to 2006. *Annals of Glaciology* **46**, 404–408 (2007).
28. Rannie, W. F. Breakup and freezeup of the Red River at Winnipeg, Manitoba Canada in the 19th century and some climatic implications. *Climatic Change* **5**, 283–296 (1983).
29. Tarand, A. & Nordli, P. The tallinn temperature series reconstructed back half a millennium by use of proxy data. *Climatic Change* **48**, 189–199 (2001).
30. Kuusisto, E. & Elo, A.-R. Lake and river ice variables as climate indicators in Northern Europe. *SIL Proceedings, 1922-2010* **27**, 2761–2764 (2000).
31. Opening and Closing of the Hudson River at Albany. in *The New York almanac and weather book* 127–128 (Mason Brothers, 1857).
32. Livingstone, D. M. Break-up dates of Alpine lakes as proxy data for local and regional mean surface air temperatures. *Climatic Change* **37**, 407–439 (1997).
33. Defila, C. & Clot, B. Phytophenological trends in Switzerland. *Int. J. Biometeorol.* **45**, 203–207 (2001).
34. Maurer, C., Koch, E., Hammer, C., Hammer, T. & Pokorny, E. BACCHUS temperature reconstruction for the period 16th to 18th centuries from Viennese and Klosterneuburg grape harvest dates. *J. Geophys. Res.* **114**, D22106 (2009).
35. Brückner, E. *Klimaschwankungen seit 1700.* Wien, Olmütz (1890).
