# Supplementary material for: A decade of cold Eurasian winters reconstructed for the early 19th century
Source: Nat Commun. 2022 Apr 19;13:2116. doi: 10.1038/s41467-022-29677-8 (PMC9019108; doi:10.1038/s41467-022-29677-8)
Supplement: Supplementary file 2 — Description of Additional Supplementary Files [file 41467_2022_29677_MOESM2_ESM.pdf]

Description of Additional Supplementary Files

File Name: Supplementary Data 1
Description: All phenological data used in this study

File Name: Supplementary Code 1
Description: Code for the reconstructions and input data (except gridded fields)
